# Open Lab Starter Kit Small Laser V2–an open source Fab lab produced laser cutter

**DOI:** 10.1016/j.ohx.2025.e00664

**Published:** 2025-06-11

**Authors:** Daniele Ingrassia, Gaia Di Martino, J.C. Mariscal-Melgar, Mohammed Omer, Liane Sayuri Honda, Luisa Lange, Marc Kohlen, Manuel Moritz, Tobias Redlich

**Affiliations:** a Institute of Production Engineering, Helmut Schmidt University, Holstenhofweg 85, 22043 Hamburg, Germany; b InMachines Ingrassia GmbH, Röntgenstraße 18A, 21493 Schwarzenbek, Germany

**Keywords:** Open Source Hardware, Laser Cutting, Open Lab Starter Kit, Open Lab, Fab Lab

## Abstract

The rise of Open Source manufacturing ecosystems is a multifaceted phenomenon, fueled by the proliferation of digital fabrication technologies, the collaborative spirit of the Open Source movement, and the creation of spaces like hackerspaces, and fab-labs. Part of the efforts is the Open Lab Starter Kit (OLSK), a set of machines designed to lower the barriers to entry for everyone. The OLSK Small Laser V2, an integral component of the OLSK, represents the second phase of design and prototyping within the scope of an Open Source Laser Cutter initiative. Being reproducibility and accessibility central goal of the OLSK, great attention was given not only to the design of the hardware, but also to the development of comprehensive documentation. The documentation, encompassing repositories and assembly manuals, assumes a pivotal role within the project framework, aiming to facilitate swift reproduction and user-friendly accessibility. Functionally, the OLSK Small Laser V2 is comparable with mainstream off-the-shelf laser cutters, demonstrating proficient cutting capabilities across commonly processed materials compatible with a 40 W CO_2_ laser cutter, such as plywood, acrylics and Medium-Density Fiberboard (MFD), with a cutting area of 400 mm x 600 mm.

## Graphical abstract

**Figure:**
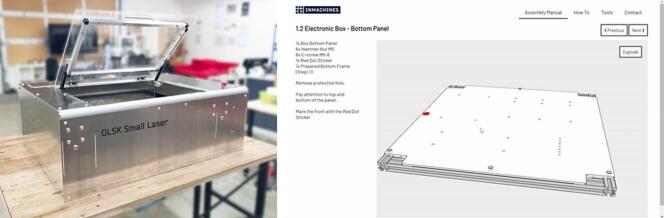

## 1 Hardware in context

### 1.1 Laser cutting

Laser cutters are CNC (Computer Numerical Control) devices that perform subtractive manufacturing through laser cutting, a method where a focused laser beam with high energy intensity melts, vaporizes, or burns the workpiece material [1,2]. This technique is favored in digital fabrication for its operational simplicity and rapid prototyping capabilities, resulting in a significant growth in its market and an increased interest for open-source variants. The most prevalent laser sources include fiber lasers, diode lasers (Nd:YVO and Nd:YAG), and gas lasers (CO2), with the selection depending on the intended application. Laser cutters are compatible with a diverse range of materials, including cellulose-based substances like wood and cardboard, various polymers, textiles, and metals such as aluminum and steel, the latter requiring specialized laser technologies. See (Table 1).

**Table 1:** OLSK Small Laser V2 Specifications.

**Specifications table**	
Hardware name	Open Lab Starter Kit Small Laser V2
Subject area	● Digital manufacturing technology ● Educational tools and Open Source alternatives to existing infrastructure
Hardware type	● Laser cutter
Closest commercial analog	FLUX Beambox Pro
Open source license	● Hardware design, CAD and PCB files, BOM, settings and other technical or design files are released under the following license: CERN Open Hardware License Version 2 Weakly Reciprocal − CERN-OHL-W ● Assembly manual, pictures, videos, presentations, description text and other type of media are released under the following license: Creative-Commons-Attribution-Share Alike 4.0 International − CC BY-SA 4.0
Cost of hardware	approximately 3.000€
Source file repository	doi: 10.5281/zenodo.10412769

### 1.2 Open source hardware

Open Source Hardware (OSH), as defined by the Open Source Hardware Association (OSHWA), is “tangible artifacts − machines, devices, or other physical things − whose designs has been released to the public in such a way that anyone can make, modify, distribute, and use those things” [3].

Open Source Hardware offers many advantages over proprietary machinery: the designs are freely accessible and often emerge from user-driven co-development. The design and building of the hardware provide comprehensive insights into the machine’s capabilities and constraints. Production is local and can virtually be carried out everywhere (even if some modifications may be necessary in terms of parts and/or materials); The Open Source movement has cultivated a community that highly values the exchange of ideas [4].

There are various small commercial laser cutters such as the Flux BeamBox Pro, GlowForge, and Mr. Beam II DreamCut; which come in different form factors and do not offer the flexibility of a freely licensed hardware project. OLSK Small Laser V2 form factor strikes a balance suitable for both digital manufacturing enthusiasts and industrial applications.

### 1.3 The Open Lab Starter Kit

In the frame of Open Source Hardware ideals, the Open Lab Starter Kit (OLSK) was born with the goal of creating a set of digital fabrication machinery to start a fully functional digital fabrication laboratory.

**Fig. 1:**
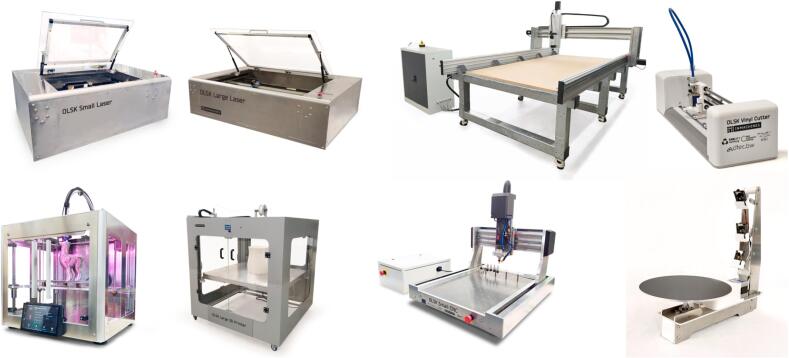
Open Lab Starter Kit: current status. From top left to bottom right: Small Laser, Large Laser, Large CNC, Vinyl Cutter, Small 3D Printer, Large 3D Printer, Small CNC, 3D Scanner.

The OLSK consists of 8 digital fabrication machines [5], as shown in Fig. 1:

● Large format laser cutter, “OLSK Large Laser”, cutting area of 1000 mm x 600 mm
● Small format laser cutter “OLSK Small Laser”, cutting area of 600 mm x 400 mm
● Large format CNC-milling machine “OLSK Large CNC”, working are of 2500 mm x 1250 mm x 300 mm
● Small format CNC-milling machine “OLSK Small CNC”, working area of 600 mm x 400 mm x 100 mm
● Large format 3D printer “OLSK Large 3D Printer”, printing area of 1000 mm x 1000 mm x 1000 mm
● Small format 3D Printer “OLSK Small 3D Printer”, printing are of 200 mm x 200 mm x 200 mm
● Vinyl cutter “OLSK Vinyl Cutter”, cutting width of 300 mm
● 3D scanner “OLSK 3D Scanner”, maximum scanning area of 300 mm x 300 mm x 300 mm

**Fig. 2:**
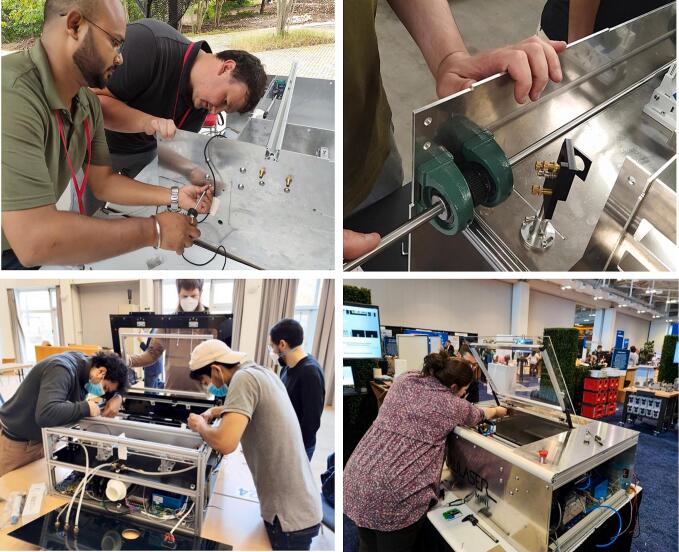
Assembly Workshops.

Most of the OLSK machines have been the object of several building workshops with the end users, as can be seen in Fig. 2; the goal of these workshops is to empower the operator by learning not only how to operate the machines, but also how they work, how to maintain them and how to repair them, if necessary.

With this aim in mind, a very important part of the Open Lab Starter Kit is the documentation, in the form assembly manual and repository.

### 1.4 Web based semi-automated assembly manual and documentation

**Fig. 3:**
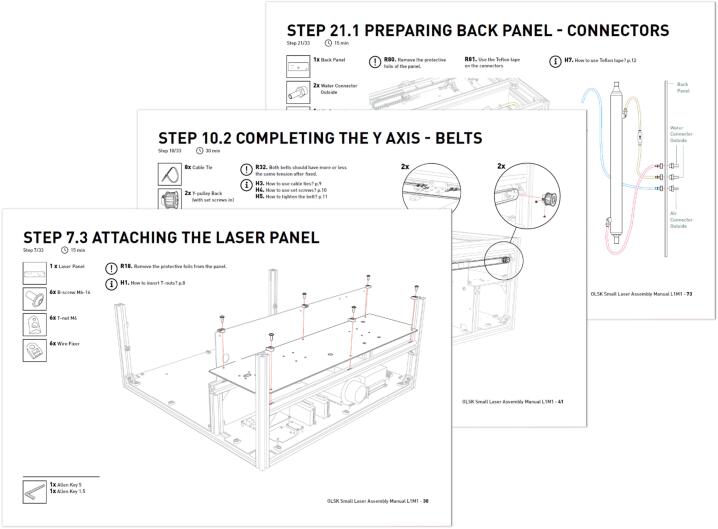
Snapshot of the OLSK Small Laser V1 manual.

**Fig. 4:**
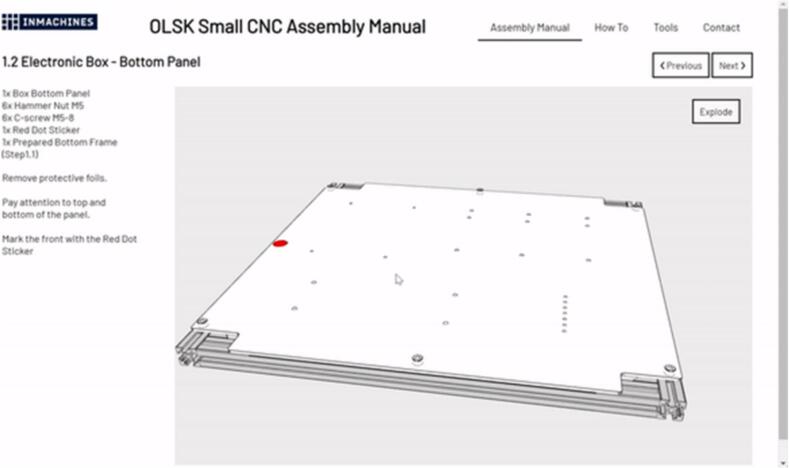
Snapshot of the web based assembly manual.

As part of the documentation of the Open Source machines, the OLSK Small Laser V1 featured an assembly manual as a static pdf showing step-by-step instructions, with clear graphic design (shown in Fig. 3), which proved efficient through multiple machine-building workshops. However, it showed itself as difficult to update and replicate. As an improved version, the OLSK Small Laser V2 assembly manual is produced and offered in a completely new manner: an online interactive 3D viewer, generated semi-automatically using the CAD file. Through this webpage, that can be seen in Fig. 4, the user is able to navigate through the steps, explode the view to understand how to assemble the parts of each step and interact with the 3D model by zooming, panning and rotating it. The process to generate it is also much simpler than its previous version. By separating the parts into steps directly in the CAD file, and exporting as a web-friendly 3D file (glTF), the online assembly manual generates the steps with its respective list and count of parts. This also makes updates in the machine version easier, since it only needs the updating of the CAD file.

## 2 Hardware description

**Fig. 5:**
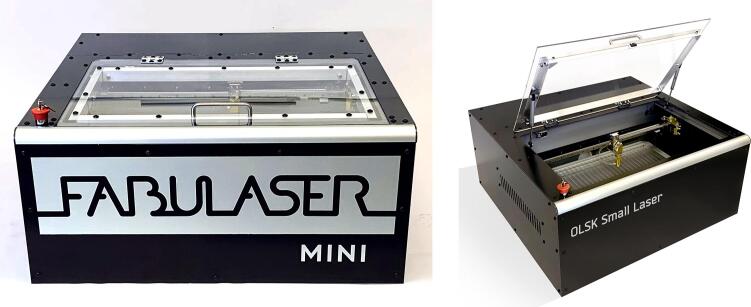
Fabulaser Mini V2 and OLSK Small Laser V1.

**Fig. 6:**
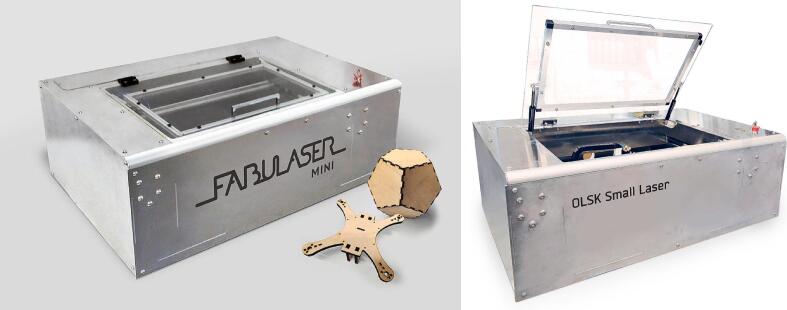
Fabulaser Mini V3 and OLSK Small Laser V2.

OLSK Small Laser V2 is an Open Source Laser Cutter, based on the Fabulaser Mini Open Source Laser Cutter, which was first developed in 2020 at InMachines by Daniele Ingrassia, as shown in Fig. 5, Fig. 6; the goal was to create an affordable, desktop laser cutter for educational purposes; the design also aimed to embrace the concept of FabLabs 2.0, where digital fabrication machines are used to replicate or create digital fabrication machines. Fabulaser Mini quickly became a staple in Nord Rhine Westphalia schools, where it was replicated in several machine-building workshops [10]. See (Table 2).

**Table 2:** OLSK Small Laser V2 technical specifications.

**OLSK Small Laser V2 Specs**	
Laser source	Water Cooled CO_2_ 40 W
Cutting area	600 mm x 400 mm
Guides	25 mm linear shaft for Y axis and 15 mm linear rail for X axis
Accuracy	± 0.05 mm
Max Speed	1000 mm/s
Frame and Housing	30 mm x30 mm Aluminum profiles and interlocked 3 mm aluminum plates
Window	6 mm Acrylic with reinforcement frame and gas springs
Bed	0.8 mm Aluminum Lamellas, adjustable in height and leveling
Sensors	Inductive Probes and Magnetic Switch
Controller	32bit iMXRT1062 (Teensy 4.1) on custom PCB shield
Firmware	grbl-HAL − iMXRT1062 driver
Cutting thickness	up to: 10 mm acrylic, 6 mm MDF, 8 mm poplar plywood
Dimensions	1161 mm x 812 mm x 390 mm (W x D x H)
Software	Visicut and an OpenSource GCode sender (e.g. Universal GCode Platform)
Accessories	Water Chiller, Air pump, Air Filter, Radial Fan

Compared to the previous version (V1), the design of the OLSK Small Laser V2 focuses on the following aspects:

● Part count reduction, consisting of about 54 % less parts (lowering the count from 1193 parts to 549 parts, by comparing BOMs)
● Possibility to manufacture the parts either in Fab Labs (e.g. using a large format CNC milling machine) or industrially (e.g. by using a fiber laser cutter)
● Possibility to use different metals to produce the housing, giving higher reproducibility possibilities depending on the local availability
● Increased ease of calibration, having fixed position for the second mirror holder.
● Integration of frame and housing into an unique rigid structure made of interlocked aluminum plates
● Scalability of working area size without changing mechanics, proved to be incrementable to at least up to 1000 mm x 600 mm (up to 66 % increase in X and 50 % increase in Y, see OLSK Large Laser V1)
● High speed motion up to 1000 mm/s, a 150 % increase compared to the 400 mm/s of the V1
● Increased safety by:
  ○ using aluminum instead of aluminum composite for the housing
  ○ having the electrics on the right side instead of under the laser tube, reducing the risk of the coolant leaking onto the electrics
  ○ using inductive probes as limit switches
  ○ separating the cable ducts for AC and DC cables

Great attention is given to implementing safety features, including several sensors which prevent the laser to be shot when the machine window is open, similarly to commercial laser cutters. This aspect is further discussed in Section 7. Validation and Characterization. Accuracy of the sensors can be found in the technical datasheets of the components.

OLSK Small Laser V2 has been used to cut cardboard, plywood up to a thickness of 8 mm, acrylic up to a thickness of 10 mm and MDF up to a thickness of 6 mm. The settings for these materials can be found in the repository as vcsettings file.

In the frame of the Open Source concept, this desktop laser cutter presents:

● Use of Standardized and Interchangeable Components: this desktop laser cutter is constructed employing components readily available in the market and designed for interchangeability. Such an approach streamlines manufacturing processes, reduces costs, and ensures broader access to essential parts, thereby promoting scalability and affordability.
● Reproducibility: the laser cutter can be produced having available a standard Fab Lab inventory or industrially with common metal working tools, such as a fiber laser cutter. Furthermore the housing can be produced of different metals depending on local availability (e.g. aluminum or steel).
● Integration of Open Source Firmware: the laser cutter operates on an Open Source firmware architecture, emphasizing transparency, and accessibility in its operational framework. This modularity allows for code examination, modification, and collaborative refinement, fostering an environment conducive to continuous improvement and customization
● Adoption of Open Source Software Suite: employing Open Source software tools such as Visicut and Universal G-code Sender (UGS) for G-code generation and machine control highlights the commitment to transparency and accessibility. These tools provide a robust platform for precise command generation and seamless control of the laser cutter, enhancing user experience and operational precision.

By leveraging standardized components, Open Source Firmware, and software tools, this laser cutter exemplifies a commitment to democratizing access to digital fabrication tools. This approach aligns with the core tenets of the Open Source movement, aiming to foster inclusivity, collaboration, and continuous advancement within the digital fabrication domain.

## 3 Design files summary

See (Table 3).

**Table 3:** Repositories list: the repositories contain all the files needed to replicate and assemble the OLSK Small Laser V2.

**Design file name**	**File type**	**Open source license**	**Location of the file**
CAD OLSK Small Laser V2	step, f3d	CERN Open Hardware Licence Version 2 Weakly Reciprocal	doi: 10.5281/zenodo.10214225
Firmware OLSK Small Laser V2	zip	Creative Commons Attribution 4.0 International	https://doi.org/10.5281/zenodo.10216168
Media OLSK Small Laser V2	png, jpg	Creative Commons Attribution 4.0 International	doi: 10.5281/zenodo.10216299
Settings OLSK Small Laser V2	vcsettings	CERN Open Hardware Licence Version 2 Weakly Reciprocal	doi: 10.5281/zenodo.10216488
Additional Documentation OLSK Small Laser V2	pdf, md	Creative Commons Attribution 4.0 International	doi: 10.5281/zenodo.10277455
Electronics OLSK Small Laser V2	pdf, md, svg, png, jpg	Creative Commons Attribution 4.0 International, CERN Open Hardware Licence Version 2 Weakly Reciprocal	doi: 10.5281/zenodo.10411270
Test Files OLSK Small Laser V2	Svg, plf, dxf	Creative Commons Attribution 4.0 International	doi: 10.5281/zenodo.10409835

## 4 Bill of materials summary

See (Table 4).

**Table 4:** Bill of Materials of OLSK Small Laser V2: Costs don’t include shipping expenses. Some electronics components for the board are not reported in the Bill of materials table.

**Name**	**Q.ty**	**Cost per Unit**	**Total Cost**	**Link**
**External Accessories**				
Power Cord (computer style) at least 2 *m*	1	7,89 €	7,89 €	https://www.amazon.de/-/en/deleyCON-Protective-Coupling-Computer-Projector-black/dp/B07Y1TJPJT/ref=sr_1_5?crid=17RRG4YAYUORE&keywords=powerd%2Bchord%2B2%2Bm&qid=1693573579&sprefix=powerd%2Bchord%2B2%2Bm%2Caps%2C186&sr=8–5&th=1
Foot switch	1	12,59 €	12,59 €	https://www.amazon.de/-/en/Brennenstuhl-Comfort-Switch-Adaptor-1508220/dp/B01JIKHVF4/ref=sr_1_6?keywords=footswitch&qid=1693574948&refinements=p_85:20943776031&rnid=20943775031&rps=1&sr=8–6&th=1
Air Filter Airmex AM03 or similar	1	525,00 €	525,00 €	https://www.airmex-nord.com/lötrauch/am-03/
Water Chiller CW-3000	1	138,87 €	138,87 €	https://www.ebay.de/itm/353776425732
Air pump Hailea ACO-318	1	44,90 €	44,90 €	https://www.ebay.de/itm/393963501960?hash=item5bba0df988:g:dH4AAOSw ∼ 1tiG ∼ Qs&amdata=enc%3AAQAIAAAA4LalGGlD%2FPDZswicWJIfNhDA2bZ3GcDeRCYN6yt8BgdPsviD8ZwdLZonM7qgk1ro8XsIqVVt4utwoSnM8CJ8deg1M5agKoK3XSnzyN9xLYa%2FgQgQxmeMXE5CoBLZCXgk%2FxCFwwXa636H%2BrTzLDmtaPOmaTyJVXTkcUkGbXQB3CLCKdZXQcrnvhMNsQvxbL72EFtF6Ox39a%2FEaIurbnVXNwTZEaIPR16LWatg3QxWLgdyju9i10VRx%2BG%2FuwIhm90Dlxi3UNV98GEBm1OUPCZeh%2BAn8z0ckOpNYO3uL%2FzF7o0e%7Ctkp%3ABFBMzNiYj8pi
Radial Fan 150 mm 150 W Neverest RV-B	1	86,37 €	86,37 €	https://www.amazon.de/Neverest-Rohrventilator-Ventilator-Leistungsstark-Radiall%C3%BCfter/dp/B07YG4DNLV/ref=asc_df_B07YG4DNLV/?tag=googshopde-21&linkCode=df0&hvadid=427689318616&hvpos=&hvnetw=g&hvrand=16873189388314541869&hvpone=&hvptwo=&hvqmt=&hvdev=c&hvdvcmdl=&hvlocint=&hvlocphy=9043534&hvtargid=pla-907659847108&psc=1&th=1&psc=1&tag=&ref=&adgrpid=97610610937&hvpone=&hvptwo=&hvadid=427689318616&hvpos=&hvnetw=g&hvrand=16873189388314541869&hvqmt=&hvdev=c&hvdvcmdl=&hvlocint=&hvlocphy=9043534&hvtargid=pla-907659847108
Water Pipe Outside inside diameter 9 mm 2 m length	2	18,25 €	18,25 €	https://www.ebay.de/itm/323585440131?var=513719600332
Water Pipe Inside Silicon 1 m 7x10mm	2	2,70 €	2,70 €	https://www.ebay.de/itm/263190368164?var=566060152729
Air pipe 4x6mm 5 *m*	1	12,98 €	12,98 €	https://www.amazon.de/gp/product/B099671H7K/ref=ppx_yo_dt_b_asin_title_o02_s03?ie=UTF8&th=1
Air pipe adapter 6–10 mm with pipe 10 mm	1	1,79 €	1,79 €	https://www.ebay.de/itm/175726486472?hash=item28ea1d63c8:g:1X8AAOSwAUlkV-r9&amdata=enc:AQAIAAAA8FNsm7bIPKO6K0mSMM0PwzeqpRsKoAEnusguVKxUMeNsnjHLMQDJhmjJzvOsa6FYDhLGCc5%2BJj1ooXyQFcFbuwyrb3lojJo7YbIa5wppz43xgYGEPchA5WKlC%2Bh179P%2FdyLrj9aGhoWam0sJXI6lZ%2BWSzumsc4Nik8afgAdbsr2o1dlAA8kEhaRnvrmIWUifB79SVIO%2F4rfwEZQ8C1WdksS%2BFUHpF7M%2B3w8LOU2wkM9jqqHvkYWvzy4%2F%2BhwbS8SmlsSBb5Z2qHuoRiTuIM5RipyDlQBdwa0fe3K5nazNymqQgavjkX2xGW2clNXXCt44RA%3D%3D|tkp:BFBMwJmGkMpi
Safety Sticker set	1	25,15 €	25,15 €	https://www.amazon.de/gp/product/B09NP6YCJL/ref=ppx_yo_dt_b_asin_title_o09_s01?ie=UTF8&th=1 https://www.amazon.de/gp/product/B07HCBKK2R/ref=ppx_yo_dt_b_asin_title_o09_s02?ie=UTF8&psc=1 https://www.amazon.de/gp/product/B09NP5HWWF/ref=ppx_yo_dt_b_asin_title_o00_s00?ie=UTF8&psc=1 https://www.ebay.de/itm/313584414991 https://www.ebay.de/itm/164935298284 https://www.ebay.de/itm/164935204648
USB Cable outside 2 m or more	1	6,27 €	6,27 €	https://www.amazon.de/-/en/StarTech-com-USB-Male-Connectors-Cable/dp/B006VYYRM6/ref=sr_1_2?crid=2TI17A4HX6XZ8&keywords=USB + cable + 2725&qid=1693576361&sprefix=usb + cable + 2725 %2Caps%2C96&sr=8–2
Exhaust hose 80 mm 1.5 *m*	2	26,90 €	26,90 €	https://www.ebay.de/itm/225730582239?var=524841485315
Hose Clamps 80 mm	4	4,00 €	16,00 €	https://www.ebay.de/itm/Schlauchschellen-Edelstahl-V2A-mit-Schneckengewinde-DIN-3017-W2-von-8 mm-210 mm/332686901917?_trkparms=ispr%3D1&hash=item4d75af429d:m:mz6IHA-rdBEK4b2fcgCT8fQ&enc=AQAEAAACcIQvEcHUrT7nmUC3yY5qbPyaBN1nJEDYW8MyypsJPgXKjzb4uel3XQiGEzY4CITgmtHiT0EfYmGFCvMrif18F%2BAx%2Fl%2FNSXWbjUQ9qTgekiDWkJx%2BuQgyIbDbO1pX6NiyjmRl0HF6WbmA6nTznzH4SWE8JVcMHzacV%2BXw%2FNLrhjcyjPh0SkfHOWzH1finbcu3G6tV37gexErMA76rgXFau46ZTaKIQDwqoqsNTkcQnQHDXTlSMj0L%2BYpGR9mWsy2aMafWC0ANdU%2BCoM4Nwo2o6bmJntNitnYNUd7%2BMsW%2FsxJ7ucuWgPh4XRKKW%2BluaxqxvtmdmkiJ%2BoGNqTE1vuWKb3nYI8MU7BS5P68Q7tvhNDptfPFCsyg7%2BxtOEHwm0UzfFp8cK6gR8m6CM8vcDNVvHz%2FPeG7MS2eTMdfieu0g9Fvze4v2NvwXGhhx3U8Wl2KZ3p00l8TMGFPen78oU1crKjM%2BWEvIvk6P%2F34aUzF%2Biq4KH1Nfwl%2FnLB%2Fa1EVsyngJ9OUW6xw9oKvgSiKnzDoHPJlMuuJBIPi0xJKK10siz9jNbyqAcL3Ag8DBAR9wK5kYI5ty2HtjLbYSEMKKbcn2Nhe%2BPvHEpUjCthPQFP89%2FJgPcmoPi1BV5rrVQl%2BP3z%2BjNpAZOY1tj1Pb4uwkfZ36LDZo39W6DQaM9rG0EP1wzmkuF1weOJrlJzKfyrrvwoStK7SPUOirsyh3ecY%2FikogwfhS0zWkTgntQrW%2BAHd7xUG679z5wTZbjn4yffLRK1vpz%2Fsm8E59vDlOc6LPxo0FafvfwXoxYAtcb12UymguR5l4nV9HVImU%2Fa5VR%2Fg3SOO9Dg%3D%3D&checksum=332686901917c7c11047856541cc85e3e361752e6538
Tesa Double Side Tape 50 mm 5 *m*	1	6,89 €	6,89 €	https://www.amazon.de/-/en/Extra-Strong-Double-Carpet-Flooring/dp/B000QB4EIM/ref=sr_1_3?crid=2ILB4AJ8Y5L4B&keywords=Tesa%2BDouble%2BSide%2BTape%2B50mm%2B5m&qid=1693577037&sprefix=tesa%2Bdouble%2Bside%2Btape%2B50mm%2B5m%2Caps%2C98&sr=8–3&th=1
Fuse 6.3A 5x20mm	2	3,99 €	3,99 €	https://www.amazon.de/-/en/Glass-5x20-522–10-P%C3%A4ckchen-0–25-1–25/dp/B01AWB3Z7Q/ref=sr_1_6?crid=1J3D8ELXHGQXF&keywords=Fuse%2B6.3A%2B5x20mm&qid=1693577134&sprefix=fuse%2B6.3a%2B5x20mm%2Caps%2C77&sr=8–6&th=1
Filter Adapter 3D Printed	1	29,99 €	29,99 €	https://www.prusa3d.com/product/prusament-pla-pristine-white-1 kg/
Radial Fan Adapter 3D Printed	2			
Focus Tool	2	Cut from “Laser Window Acrylic 750x600 6 mm” left-overs		
Rubber Rings 20 mm	2	1,95 €	3,90 €	https://www.ebay.de/itm/185767312676?hash=item2b40983d24:g:IZQAAOSwerZdzVit&amdata=enc:AQAIAAAA4CXMnpOvhkR%2F3ASFc%2BQbmQDkWj9FJ9gHk%2FEoudgl453pz0LwtcqVEgnZORbNd9IpRMf7Vc6WjpQweYY89eaFuZLDXC78EOPDgUm1USZjw%2FEMS8d%2Ft0fhrj9Xe%2B89mvKb4KRquEBbG7eVcf%2F0tsjxFFohUuKHMq0Imc01tgLMqMHPkJ08ulYjk%2FJS03pVpkcKlul%2FEQ62JSRd6PSvS6Yz47bBXVEj2MvLm6%2FPXwlOSolUvAtVdTikUXXABX0NFMGhiI1zLL4uN5W1RPxwTdh3yRq99A2N%2BjBbjT5r23oqUXe3|tkp:Bk9SR5be7pHKYg
Optical Paper	1	8,90 €	8,90 €	https://www.amazon.de/Lens-Aid-Lens-Paper-Cleaning-Tear-Resistant/dp/B08FDLJ93Q/ref=sr_1_{1s}spa?keywords=lens + cleaning + paper&qid=1693578456&sprefix=lens + cleaning + pa%2Caps%2C86&sr=8–1-spons&sp_csd=d2lkZ2V0TmFtZT1zcF9hdGY&psc=1
Wago Connectors Set	1	28,48 €	28,48 €	https://www.amazon.de/-/en/Wago-clamps-pieces-assortment-consists/dp/B0846QDN1Y/ref=sr_1_17?crid=2SMVHU9N0BTMZ&keywords=wago + connector&qid=1693578690&refinements=p_85:20943776031&rnid=20943775031&rps=1&sprefix=wago + connte%2Caps%2C97&sr=8–17
Cable Ties min. 150 mm 100 pieces	1	4,69 €	4,69 €	https://www.amazon.de/-/en/mumbi-Cable-UV-Resistant-Protection-Professional/dp/B0BT4X7SM8/ref=sr_1_5?crid=35PLJBJ5EYI1S&keywords=cable + ties + 150 mm + x + 2.5&qid=1693578774&sprefix=cable + ties + 150 mm + x + 2.5 %2Caps%2C75&sr=8–5
X Axis				
Ball Bearing 6801	4	11,60 €	11,60 €	https://www.ebay.de/itm/261661289497?hash=item3cec3a6c19:g:T40AAOSw2XFUZRiZ&amdata=enc:AQAIAAAA4BWdh%2FHABLUHJxBlqjHun007u0u3QzbWSeEw%2FUdLRjo2GJkBYozjSGJfAwWsjD5489uiqOcF%2BEObRzFDNVAr%2FC3n5af6Mq2grbhwawWt4wKRYL9QH6nBhDwaSVla1pzm3haOEYKe5nGOelDqsrIetVcoLbQWBOupO7bUXDmIO%2F4W1O%2BeVE7%2B51BWgEHP0e9xhy1QOjmgNk99KR3ZCnjhML6AXqi3Lp9KfemnaSRSZnyKNunsZgWiuUKyBRCu7U21G1QCAtFrfz7w0gs3rXbkCH5XNPNkHi0wAhIg7RpsqJhW|tkp:BFBM9vz4kspi
Stepper Motor 24HS39-4204D with Cable 4x0.5 mm 150 cm ground shield	1	46,35 €	46,35 €	https://www.omc-stepperonline.com/de/search?search=24HS39-4204D&description=true
		9,00 €	0,00 €	https://www.ebay.de/itm/122857362393?hash=item1c9ade93d9:g:8qwAAOSw9gRaMAwX&amdata=enc%3AAQAIAAAA0L5FmiooBwUx9l0tSh%2Bnng14D5%2BDe5vE2CNBy48dGPFdJHTUxtnEw%2BNh0y%2Frvsk5fs3zZdZJdclHoH09bVA7L0%2FCF2BB1pNs13rMoQpzyOCXjLSVmuuhWmzPHLF%2FEiXnMMZ9jrn6scu03pjsp%2BuN94EX1qV1121ecgMFLbMC9C4IXJYjTbFjHlrFjphzi5Ec5PYBXYjMn4o%2FV9I%2BsF2UNM432wuQwSJgOKbH9s%2FnIZKk2bYhnzMqzjMEDp3lSvWACCG3LTv6UAk%2FJ8sTFubP7OU%3D%7Ctkp%3ABk9SR_T2npXKYg
X Plate Aluminum 8 mm	1	102,45 €	102,45 €	https://shop.ibl-raimund.de/Alu/Platten/Maschinenbau-AlMg3/Aluminiumplatte-EN-AW-AlMg3-5754-Staerke-8-mm.html
X Belt HTD 5 M 10 2075 mm	1	61,97 €	61,97 €	https://www.zahnriemen24.de/a/40041-zahnriemen-meterware-5 m-pu?breite=10&offenv=2&polyamidgewebe=1&laenge=2075&quantity=1
Y Linear Bearing SCE25UU Left	1	12,21 €	12,21 €	https://www.dold-mechatronik.de/Linearlager-25 mm-SCE25UU
Y Linear Bearing SCE25UU Right	1	12,21 €	12,21 €	https://www.dold-mechatronik.de/Linearlager-25 mm-SCE25UU
X Linear Guide HGR15 T 850 mm	1	48,40 €	48,40 €	https://www.cnc-store.eu/en/guida-hiwin-hgr15-t-c-fori-ciechi.html?search=00000605
X Left Pulley HTD 5 M 28 T 9 mm	1	9,85 €	9,85 €	https://www.zahnriemen24.de/a/40140-zahnscheiben-5 m?zaehnezahl=28&riemenbreite=9&bohrung=1&vorbohrungspannsatz=1&quantity=1
X Right Pulley HTD 5 M 28 T 9 mm	1	9,85 €	9,85 €	https://www.zahnriemen24.de/a/40140-zahnscheiben-5 m?zaehnezahl=28&riemenbreite=9&bohrung=1&vorbohrungspannsatz=1&quantity=1
X Shaft 12 mm 35 mm	1	1,60 €	1,60 €	https://www.dold-mechatronik.de/Praezisionswelle-12 mm-h6-geschliffen-und-gehaertet-100 mm
Y Axis				
Y Shaft 25 mm 800 mm Tapped M12	2	30,60 €	61,20 €	https://www.dold-mechatronik.de/Praezisionswelle-25 mm-h6-geschliffen-gehaertet-800 mm-mit-Gewindebohrungen-M12x30
Y Belt HTD 5 M 15 mm 2000 mm cut to 1525 mm	2	31,65 €	63,30 €	https://www.zahnriemen24.de/a/40008-zahnriemen-5 m?breite=15&laenge=2000&quantity=1
Stepper Motor 24HS39-4204D with Cable 4x0.5 mm 100 cm ground shield	1	See “Stepper Motor 24HS39-4204D with Cable 4x0.5 mm 150 cm ground shield”		
Shaft 12 mm 150 mm	1	2,40 €	2,40 €	https://www.dold-mechatronik.de/Praezisionswelle-12 mm-h6-geschliffen-und-gehaertet-150 mm
Shaft 12 mm 100 mm	1	1,60 €	1,60 €	https://www.dold-mechatronik.de/Praezisionswelle-12 mm-h6-geschliffen-und-gehaertet-100 mm
Shaft 12 mm 1000 mm	1	16,00 €	16,00 €	https://www.dold-mechatronik.de/Praezisionswelle-12 mm-h6-geschliffen-und-gehaertet-1000 mm
Y Motor Holder 3D Printed With Inserts M8	1	9,99 €	9,99 €	https://www.amazon.de/-/en/threaded-RX-M8x12-7-Threaded-Press-fit-ultrasound/dp/B085X28168/ref=sr_1_1?crid=2OR4EDMQW04R0&keywords=inserts%2Bm8%2B20%2Bruthex&qid=1693581386&sprefix=inserts%2Bm8%2B20%2Bruthex%2Caps%2C248&sr=8–1&th=1
		See “Filter Adapter 3D Printed”		
Y Belt Attacher Aluminum 6 mm	4	See “Head Holder Aluminum 6 mm”		
Y Pulley HTD 5 M 26 T 9 mm	1	9,30 €	9,30 €	https://www.zahnriemen24.de/a/40140-zahnscheiben-5 m?zaehnezahl=26&riemenbreite=9&bohrung=1&vorbohrungspannsatz=1&quantity=1
Y Pulley HTD 5 M 16 T 9 mm	1	5,35 €	5,35 €	https://www.zahnriemen24.de/a/40140-zahnscheiben-5 m?zaehnezahl=16&riemenbreite=9&bohrung=1&vorbohrungspannsatz=1&quantity=1
Y Pulley HTD 5 M 26 T 15 mm	4	11,20 €	44,80 €	https://www.zahnriemen24.de/a/40140-zahnscheiben-5 m?zaehnezahl=26&riemenbreite=15&bohrung=1&vorbohrungspannsatz=1&quantity=1
Y Motor Belt HTD 5 M 9 mm 305 T	1	8,10 €	8,10 €	https://www.zahnriemen24.de/a/40008-zahnriemen-5 m?breite=9&laenge=305&quantity=1
Pillow Block 12 mm UCPA201	8	9,90 €	79,20 €	https://www.ebay.de/itm/331354351426?var=540546770082
Housing				
Front Panel Aluminum 4 mm	1	72,62 €	72,62 €	https://shop.ibl-raimund.de/Alu/Platten/Maschinenbau-AlMg3/Aluminiumblech-EN-AW-AlMg3-5754-Staerke-4-mm-oxid.html
Back Panel Aluminum 4 mm	1	72,62 €	72,62 €	https://shop.ibl-raimund.de/Alu/Platten/Maschinenbau-AlMg3/Aluminiumblech-EN-AW-AlMg3-5754-Staerke-4-mm-oxid.html
Right Panel Aluminum 3 mm	1	400,21 €	400,21 €	https://shop.ibl-raimund.de/Alu/Platten/Maschinenbau-AlMg3/Aluminiumblech-EN-AW-AlMg3-5754-Staerke-3-mm.html
Separator Panel Aluminum 3 mm	1			
Laser Panel Aluminum 3 mm	1			
Cover Panel Aluminum 3 mm	1			
Window Spacer Left Aluminum 3 mm	1			
Window Spacer Back Aluminum 3 mm	1			
Window Spacer Right Aluminum 3 mm	1			
Sub Window Front Aluminum 3 mm	1			
Sub Window Right Aluminum 3 mm	1			
Sub Window Left Aluminum 3 mm	1			
Sub Window Back Aluminum 3 mm	1			
Inner Panel Left Aluminum 3 mm	1			
Inner Panel Right Aluminum 3 mm	1			
Left Panel Aluminum 3 mm	1			
Top Panel Aluminum 3 mm	1			
Bottom Panel Aluminum 3 mm	1			
Front Support 3D Printed	1	See “Filter Adapter 3D Printed”		
Top Aluminum Profile 30x60 Nut 8 800 mm	2	15,42 €	30,84 €	https://www.dold-mechatronik.de/Aluminiumprofil-30x60L-B-Typ-Nut-8–149 kg-m-Zuschnitt-50–6000 mm
Bottom Aluminum Profile 30x30 Nut 8 800 mm	3	8,97 €	26,91 €	https://www.dold-mechatronik.de/Aluminiumprofil-30x30L-B-Typ-Nut-8–084 kg-m-Zuschnitt-50–6000 mm
Fillet Aluminum Profile 30x30 Nut 8 1145 mm	1	12,27 €	12,27 €	https://www.dold-mechatronik.de/Designprofil-Aluminiumprofil-30x30L-Radius-25-B-Typ-Nut-8–078 kg-m-Zuschnitt-50–6000 mm
Front Bracket 30x20x3 200 mm	1	2,99 €	2,99 €	https://www.ebay.de/itm/221833745629?var=520715417225
Exhaust Adapter 3D Printed	1	See “Filter Adapter 3D Printed”		
Water Connector 9–9 mm Screw 11.7 mm	2	6,49 €	12,98 €	https://www.amazon.de/gp/product/B08L6V4RFH/ref=ppx_yo_dt_b_asin_title_o02_s04?ie=UTF8&th=1
Air Connector Press Fit 6–6 mm	1	See “Air pipe 4x6mm 5 m”		
**Bed**				
Aluminum Profile 40x40 Nut 8 500 mm	2	10,25 €	20,50 €	https://www.dold-mechatronik.de/Aluminiumprofil-40x40L-I-Typ-Nut-8-leicht-176 kg-m-Zuschnitt-50–6000 mm
Bed Bracket 40x40x3 700 mm	2	5,23 €	10,46 €	https://www.ebay.de/itm/321496185505?var=510380657506
Bed Lamella Aluminum 35x40x0.8 500 mm	16	5,94 €	95,04 €	https://designbleche-shop.de/de/2000 mm-aluminim-winkel-silber-natur-eloxiert-246–259.html
**Window**				
Window Front Profile Aluminum 20x20 599 mm	1	3,29 €	3,29 €	https://www.ebay.de/itm/222004924597?var=520876544706
Window Left Profile Aluminum 20x20 380 mm	1	2,99 €	2,99 €	https://www.ebay.de/itm/222004924597?var=520876544706
Window Right Profile Aluminum 20x20 380 mm	1	2,99 €	2,99 €	https://www.ebay.de/itm/222004924597?var=520876544706
Window Back Profile Aluminum 20x20 599 mm	1	3,29 €	3,29 €	https://www.ebay.de/itm/222004924597?var=520876544706
Laser Window Acrylic 750x600 6 mm	1	49,15 €	49,15 €	https://www.ebay.de/itm/265727576896?hash=item3dde990b40:g:VW4AAOSw4BZf3kcy
Hinge GN 237-ZD-60–60-A-SW	2	21,51 €	43,02 €	https://www.amazon.de/gp/product/B01M4O6HQ8/ref=ppx_yo_dt_b_asin_title_o02_s05?ie=UTF8&th=1
Window Connector 20x20 3D Printed	2	See “Filter Adapter 3D Printed”		
Piston Support Right 3D Printed	1	See “Filter Adapter 3D Printed”		
Piston Support Left 3D Printed	1	See “Filter Adapter 3D Printed”		
Piston 100 N Length 250 Travel 80 mm	2		0,00 €	
Window Handler 192 mm	1	2,81 €	2,81 €	https://www.ebay.de/itm/352012618682?var=621522181952
Window Angle Press Fit 20x20	4		0,00 €	
**Electrics / Electronics**				
USB Cable Inside	1	2,29 €	2,29 €	https://www.ebay.de/itm/175584238791
Resistor 1 K	3	6,54 €	6,54 €	https://www.amazon.de/-/en/gp/product/B07KX37J53/ref=ppx_yo_dt_b_search_asin_title?ie=UTF8&psc=1
Cable Duct 25x40 700 mm	2	9,42 €	9,42 €	https://www.ebay.de/itm/175057818155
Stepper Motor Driver DM556T	2	19,99 €	39,98 €	https://www.amazon.de/gp/product/B093LF6LPG/ref=ppx_yo_dt_b_asin_title_o01_s00?ie=UTF8&psc=1
Motor Power Supply RSP-500-48A	1	95,57 €	95,57 €	https://www.omc-stepperonline.com/rsp-500–48-mean-well-504w-48vdc-10-5a-115-230vac-single-output-with-pfc-function-rsp-500–48
Inductive Probe LJ12A3-4-Z/AY with cable 3x0.25 mm 320 cm + 140 cm ground shield	2	5,99 €	11,98 €	https://www.ebay.de/itm/123250430545
		4,99 €	0,00 €	https://www.ebay.de/itm/183566517594?var=690941097742
Window Sensor PS-3150 with cable 2x0.25 160 cm ground shield	1	4,77 €	4,77 €	https://www.ebay.de/itm/174939181346
Window Magnet PS-3150	1	1,10 €	2,20 €	https://www.ebay.de/itm/283736151360?hash=item420ffe1540:g:srwAAOSwjzleFeTW
Logic Power Supply LRS-100–24	1	22,89 €	22,89 €	https://www.ebay.de/itm/162929175682
Laser Power Supply MYJG50W	1	45,99 €	45,99 €	https://www.ebay.de/itm/403959124666?var=673838757648
Window Sensor Holder 3D Printed	1	See “Filter Adapter 3D Printed”		
Cable Chain inner size 25x18 outer size 35x23 1000 mm	1	15,00 €	15,00 €	https://www.ebay.de/itm/383608628106?var=652004675165
Power Plug 48x28x27mm M4	1	11,19 €	11,19 €	https://www.amazon.de/-/en/gp/product/B0BLJY9C2V/ref=ppx_yo_dt_b_search_asin_title?ie=UTF8&psc=1
Cable Chain inner size 15x15 outer size 24x20 2000 mm	1	29,98 €	29,98 €	https://www.ebay.de/itm/383608628106?var=652004675178
X Chain Spacer 3D Printed	1	See “Filter Adapter 3D Printed”		
Y Endstop Holder 3D Printed	1	See “Filter Adapter 3D Printed”		
X Endstop Holder Pom 6 mm	1	3,51 €	3,51 €	https://www.ebay.de/itm/333273781462?hash=item4d98aa54d6:g:1kMAAOSwLcBia62z&amdata=enc%3AAQAIAAAA4Os4HXXIS%2FrygkMMw3jrnkJ%2BIkb4XBSmDsOAZuYY7YzBJ4I3cU7CyDuz0IAdFuiES5muqpFOKeW2QfvM3SFeuDKzRt6zFw%2Ftbzmt18oTgvcN3jvEy3b2keOCtve0QH0bSUNXUPyePOwzUnN%2FlaoN5C%2FQWeNjgADnvBIPEIqq1ml9DGF8uz3ytq8xBqH%2BuqQ1YcGh39ZeyIao%2Fui%2BQz8A1a5YvqWmHofH%2B2Gakfr1orSB2UOW7bKL5m%2FDhRAsN8h%2Bz7h9M7B%2Fx4fnFu%2BOi1eHjUZ83TtDw7%2FGt1W0UISAzpT4%7Ctkp%3ABk9SR-zioqf-Yg
Y Cable Organizer 3D Printed	1	See “Filter Adapter 3D Printed”		
Chiller Connector 3 Pole with 20 cm 2x0.25 mm cable no shield	1	16,37 €	16,37 €	https://www.ebay.de/itm/334693663378?hash=item4ded4c0292:g:ciwAAOSwB4NWwImU&amdata=enc:AQAIAAAA0MneTKIiDNeIgh9tBKWYm8lTAHNxY0VMiciMwFy%2BeaSvuKE90Vq1CeIkeOiTwf%2BsQieRyw0S5kqN79oxvOWnRSIonwJ%2B4BHAkIKGdu2prdeatc8vx9s8qIIymWwpNqQtoNiAmNDZTmk2ivQIWPmOHUxETXEXYQxr3NGRMzwSZOAQSSNqnFK0OsR6cLEvfKgbDKTAxs0VlheF%2BBVTQHUOJs5ak8tsMbcj%2FXQnYKRgHweUAGJLYX1V7AypHQaSN20MRs3XlUxcXc%2BOV2gDifZjJ8Y%3D|tkp:Bk9SR-LPxKn-Yg
Emergency Button With Key AS22-B142	1	5,99 €	5,99 €	https://www.ebay.de/itm/281817794707?var=585592937356
M12x1.5 Cable gland v1	2		0,00 €	
Controller PCB (with Teensy 4.1 PJRC Development Board 600 MHz ARM Cortex-7 and ATtiny85-20PU ATMEL-AVR-RISC-µC 20 MHz 8 KB FLASH 2,7–5,5V DIP8)	1	14,20 €	14,20 €	https://www.amazon.de/-/en/sourcing-Copper-Circuit-Laminate-Projects/dp/B09NJLP7T8/ref=sr_1_5?crid=OZVHYKMEITNO&keywords=platinen + copper&qid=1700659657&sprefix=platinen + copper%2Caps%2C100&sr=8–5
		2,89 €	2,89 €	https://www.ebay.de/itm/335013554289?hash=item4e005d2871:g:1qQAAOSw4eNk8yWB&amdata=enc:AQAIAAABAIYevN60KZHPpwvkdZqefOZvub7Q%2FPSSpA63PV48yoQcVTqxeabYgisCq9MJT1jUcwsTkcm0EsR1d5AMgfsyyprZTNyHwxi6xJ3BA1MX1Yhci9rchJXeNC1VL%2FjCTUn4mKymXADdTFel5REM9qF1xcVSXIi8ebVPHDJwL959NtoNNm0xWap1BgK8IBMbwtfxNLx7EgU3uDB02rU5QyoW677YAvo2fA%2Bgvd7rQd48XwNkdh7RyPi9ukYmG5ReajekV0Zc%2FXkvfLUPeFz%2F%2Bw2FdkijizMf03XPlVxgMoki1BwPoOaJAWCTBw2ZY5sun4QP8ZagdHuiU9PyIxYx3EkNGiM%3D|tkp:BFBMzOun8_5i
		55,49 €	55,49 €	sps:xlink.href::https://www.ebay.de/itm/285549593143?hash=item427c150237:g:∼ycAAOSwA ∼ dlWs5U||xlink.type::simple
Pipe Holder M6 10 mm	2	6,99 €	13,98 €	https://www.amazon.de/gp/product/B07VCJWP99/ref=ppx_yo_dt_b_asin_title_o09_s00?ie=UTF8&psc=1
Wire Fixer	6	See “Filter Adapter 3D Printed”		
Driver Power Cable 2x1mm 60 cm ground shield	2	1,38 €	4,14 €	https://www.ebay.de/itm/354345656132?var=623746616471
Controller Power Cable 2x1mm 70 cm ground shield	1			
Driver Logic Cable 4x0.5 mm 40 cm ground shield	2	1,60 €	3,20 €	https://www.ebay.de/itm/354345656132?var=623746616471
Laser Logic Cable 3x0.25 90 cm ground shield	1	1,17 €	1,17 €	https://www.ebay.de/itm/354345656132?var=623746616471
Chiller Cable 2 cores min. outer diameter 6.5 mm 2 m with 2 x chiller connectors	1	See “Chiller Connector 3 Pole with 20 cm 2x0.25 mm cable no shield (3 cores used)”		
Mains Cable AC 3x1.5 mm 25 cm	1	11,00 €	11,00 €	https://www.ebay.de/itm/272663902835?hash=item3f7c08fa73:g:aJ8AAOSwt ∼ 5kcG3R&amdata=enc%3AAQAIAAABIFMIK1Z%2F%2Bq%2F%2Bxpf0BrNosj5UWHHlhZtsF1M96vmwwApZvgRCgSkveJ6wnpsTNhFnk2xVIHa4eTqQB%2BqZTRIZXEKgnx%2FGqjWEqjO%2FWIOhXz2OtkGPmwUak3h%2FO9xzJYWpsbZrkwipB0GFk5PIq7igfP%2FlVB12U3NddqLBBZYWSr%2FGQsGC9nPYc8fzKnHiqQ3U8nS7PaQLNC4cm8zIjtxt6lQqz76jzODleBlaO%2F%2BAU4%2FqzBqUyvNsYwNeIUeZ8bZE9dpPUI%2BZBKO945aLr9lQbEGkX0MhB0CRc8pK7bVUJCO7ST96L0QBiiO3ogylA8vhYBLv%2BZIJFlMubsDbz7jki8HOAY1TM3cEKdU9l5a2V%2BzSw28P3RJIOjaImutw7P4Exw%3D%3D%7Ctkp%3ABFBMmJWBqP5i https://www.ebay.de/itm/353772841189?var=623138129547
AC Jumper 1x1.5 mm 8 cm	1			
Earth Cable AC 1x1.5 mm 15 cm	1			
AC Power Cable 3x1.5 mm 20 cm	3	12,85 €	12,85 €	
AC Extension Cable 3x1.5 mm 25 cm	1			
Emergency Button Cable 2x1.5 mm 110 cm	1			
Led Strip 24 V Warm White 40 cm with cable 2x0.75 70 cm + 2x0.75 230 cm	2	22,99 €	22,99 €	https://www.amazon.de/gp/product/B08XLSW8C5/ref=ppx_yo_dt_b_asin_title_o01_s01?ie=UTF8&th=1
**Laser**				
Laser connector (screw terminal mammut) 2 pos	2	3,79 €	7,58 €	https://www.amazon.de/-/en/Connex-COXB370390-Terminal-Blocks-Pack/dp/B09446BM2V/ref=sr_1_36?keywords=terminal%2Bblock%2Bconnector&qid=1700582954&sr=8–36&th=1
Laser Mirror 25 mm	3	32,90 €	98,70 €	https://www.amazon.de/gp/product/B07MJLKBPM/ref=ppx_yo_dt_b_asin_title_o02_s04?ie=UTF8&th=1
Laser Lens 20 mm 50.8 mm	1	25,50 €	25,50 €	https://www.amazon.de/gp/product/B01EMZD262/ref=ppx_yo_dt_b_asin_title_o02_s04?ie=UTF8&th=1
First Mirror Holder 25 mm	1	27,20 €	27,20 €	https://www.aliexpress.com/item/1005005815933873.html?spm=a2g0o.productlist.seoads.9.32c52bd51Bx8kx&p4p_pvid=202311280845333398487926063440001537061_5&s=p
Laser Tube Holder 45–80 mm	2	24,19 €	24,19 €	https://www.ebay.de/itm/404517308384?hash=item5e2f1c47e0:g:taMAAOSw5ndlE7t2&amdata=enc%3AAQAIAAAA4GIhb5cXwLbbd8wkLSkGLdvDv7b3TU%2BJFtv%2FxC8Y%2BwjE27XfcVy1jnEcD0CFyzxHlV2pc0EgmGXZMSk2xdCTyOt5amj3LclVRHlPpXq4oIthy%2BfevqrM%2FLG0DZEeZTTC6XB5EDP2GycoLlOhEwypGlFuAOLJzAlX%2B7EJs8ZhWtIce6RbbPfJBGjFMvNWGWPzMYsWWLLhyfh0qbR0C2bUFuSKMfUeqyvMthZRI%2Fz7Slb7mlyw%2BEvhFqfVPKZwqoFiyQGc6N%2FHq%2Fvwaz%2F8tok2nnbcA39BCXpxLp9W8ryZnIYc%7Ctkp%3ABFBM_reVq_5i
Laser Tube CO2 40 W 50x720mm	1	66,99 €	66,99 €	https://www.ebay.de/itm/394206663693?var=662721051699
Second Mirror Holder 25 mm	1	28,87 €	28,87 €	https://www.aliexpress.com/item/1005005816007565.html?spm=a2g0o.store_pc_allProduct.8148356.1.28ae364dNihHnH&pdp_npi=4 %40dis%21EUR%21 %E2%82 %AC%2028 %2C87%21 %E2%82 %AC%2017 %2C32%21 %21 %21221.05 %21132.63 %21 %4021038edc17011901313181139e8a36%2112000034449688823 %21sh%21DE%210 %21
Laser Mirror Spacer 3D Printed	1	See “Filter Adapter 3D Printed”		
Laser Head Mirror 25 Lens 20	1	25,26 €	25,26 €	https://www.amazon.de/-/en/gp/product/B09R28KYRQ/ref=ppx_yo_dt_b_search_asin_title?ie=UTF8&th=1
**Head**				
Head Holder Aluminum 6 mm	1	24,83 €	24,83 €	https://shop.ibl-raimund.de/index.php?cl=details&cnid=b18cba9430985474665e0bf701706904&anid=9a3c30ecccb7beda26491722425ea08a&listtype=list&&sid=7q42cd25eckae6iuhbavtaeoc2
X Carriage QHH15CA1T1200Z0C	1	26,23 €	26,23 €	https://www.cnc-store.eu/en/bearing-block-hgh15cazac.html?search=00000487
Belt Attacher Aluminum 6 mm	1	See “Head Holder Aluminum 6 mm”		
**Fasteners**				
ISO 7380 M10 12 mm	16	14,84 €	14,84 €	https://www.ebay.de/itm/371775081777?var=640748904385
ISO 7380 M8 10 mm	38	9,03 €	9,03 €	https://www.ebay.de/itm/126065852743?hash=item1d5a1c4147:g:JS8AAOSwtQhk6JY∼
ISO 7380 M5 25 mm	4	4,33 €	4,33 €	https://www.ebay.de/itm/126065852743?hash=item1d5a1c4147:g:JS8AAOSwtQhk6JY∼
ISO 7380 M4 10 mm	37	4,90 €	4,90 €	https://www.ebay.de/itm/162259230026?var=461236651055
ISO 7380 M12 20 mm	4	11,32 €	11,32 €	https://malimo24.de/ISO-7380-Linsenkopfschrauben-Edelstahl-A2-M12x20-mm-25-Stueck
ISO 7380 M8 20 mm	24	6,38 €	6,38 €	https://www.ebay.de/itm/126065852743?hash=item1d5a1c4147:g:JS8AAOSwtQhk6JY∼
DIN 912 M5 12 mm	22	5,33 €	5,33 €	https://www.ebay.de/itm/125958291643?hash=item1d53b300bb:g:TPsAAOSwFg1kirOD
DIN 912 M6 16 mm	8	4,97 €	4,97 €	https://www.ebay.de/itm/125958291643?hash=item1d53b300bb:g:TPsAAOSwFg1kirOD|
DIN 912 M5 16 mm	8	4,71 €	4,71 €	https://www.ebay.de/itm/125958291643?hash=item1d53b300bb:g:TPsAAOSwFg1kirOD
DIN 912 M4 16 mm	4	4,73 €	4,73 €	https://www.ebay.de/itm/125958291643?hash=item1d53b300bb:g:TPsAAOSwFg1kirOD
DIN 912 M3 8 mm	6	4,66 €	4,66 €	https://www.ebay.de/itm/125958291643?hash=item1d53b300bb:g:TPsAAOSwFg1kirOD
DIN985 M8	34	6,63 €	6,63 €	https://www.ebay.de/itm/124269120502?hash=item1cef044bf6:g:IMgAAOSwEaZfMp4∼
DIN 985 M5	8	4,36 €	4,36 €	https://www.ebay.de/itm/124269120502?hash=item1cef044bf6:g:IMgAAOSwEaZfMp4∼
Plastic DIN 7985 M3 8 mm	6	11,29 €	11,29 €	https://www.amazon.de/-/en/gp/product/B09MZ2JZY8/ref=ppx_yo_dt_b_search_asin_title?ie=UTF8&psc=1
Nut 8 M8	16	3,73 €	3,73 €	https://www.motedis.com/en/T-nut-guided-I-type-slot-8-M8
Nut 6 M5	3	0,96 €	0,96 €	https://www.motedis.com/en/T-nut-with-spring-ball-with-guidance-I-Type-slot-6-M5
Nut 6 M4	2	0,74 €	0,74 €	https://www.motedis.com/en/T-nut-with-spring-ball-with-guidance-I-Type-slot-6-M4
DIN 125 M8	2	3,58 €	3,58 €	https://www.ebay.de/itm/124088182306?hash=item1ce43b6622:g:HTEAAOSwNkZfM44-
Washer M4	4	0,37	1,48 €	https://www.ebay.de/itm/124088182306?hash=item1ce43b6622:g:HTEAAOSwNkZfM44-
ISO 7380 M8 55 mm	2	10,55 €	10,55 €	https://www.ebay.de/itm/371775081777?var=640748904428
DIN 985 M6	12	5,28 €	5,28 €	https://www.ebay.de/itm/124269120502?hash=item1cef044bf6:g:IMgAAOSwEaZfMp4∼
ISO 7380 M6 35 mm	9	5,00 €	5,00 €	https://www.ebay.de/itm/126065852743?hash=item1d5a1c4147:g:JS8AAOSwtQhk6JY∼
DIN 912 M3 12 mm	4	4,55 €	4,55 €	https://www.ebay.de/itm/125958291643?hash=item1d53b300bb:g:TPsAAOSwFg1kirOD
DIN 985 M3	4	3,21 €	3,21 €	https://www.ebay.de/itm/124269120502?hash=item1cef044bf6:g:IMgAAOSwEaZfMp4∼
DIN 7991 M8 25 mm	4	4,50 €	4,50 €	https://www.ebay.de/itm/125958341983?hash=item1d53b3c55f:g:IC8AAOSwHWtlSJ-k
DIN 9021 M8 Washer	4	0,62 €	0,62 €	https://www.motedis.com/en/Washer-DIN-9021-for-M3-M4-M5-M6-M8-M10-and-M12
DIN 912 M4 35 mm	5	4,76 €	4,76 €	https://www.ebay.de/itm/125958291643?hash=item1d53b300bb:g:TPsAAOSwFg1kirOD
ISO 7380 M8 30 mm	6	5,29 €	5,29 €	https://www.ebay.de/itm/126065852743?hash=item1d5a1c4147:g:JS8AAOSwtQhk6JY∼
Standoff M8 15–8 Male-Female	2	12,20 €	12,20 €	https://www.ebay.de/itm/313859084521?var=612592197547
DIN 912 M4 12 mm	9	4,60 €	4,60 €	https://www.ebay.de/itm/125958291643?hash=item1d53b300bb:g:TPsAAOSwFg1kirOD
DIN 912 M4 10 mm	8	4,72 €	4,72 €	https://www.ebay.de/itm/125958291643?hash=item1d53b300bb:g:TPsAAOSwFg1kirOD
DIN 912 M8 130 mm	4	6,79 €	6,79 €	https://www.ebay.de/itm/126040022548?hash=item1d58921e14:g:uD0AAOSwhSpky1g0
DIN 912 M3 6 mm	5	4,73 €	4,73 €	https://www.ebay.de/itm/125958291643?hash=item1d53b300bb:g:TPsAAOSwFg1kirOD
DIN 7991 M8 50 mm	4	4,80 €	4,80 €	https://www.ebay.de/itm/125958341983?hash=item1d53b3c55f:g:IC8AAOSwHWtlSJ-k
DIN 912 M5 30 mm	4	5,09 €	5,09 €	https://www.ebay.de/itm/125958291643?hash=item1d53b300bb:g:TPsAAOSwFg1kirOD
ISO 7380 M6 16 mm	2	4,31 €	4,31 €	https://www.ebay.de/itm/126065852743?hash=item1d5a1c4147:g:JS8AAOSwtQhk6JY∼
ISO 7380 M8 16 mm	2	4,42 €	4,42 €	https://www.ebay.de/itm/126065852743?hash=item1d5a1c4147:g:JS8AAOSwtQhk6JY∼
Standoff Male Female M3 20 mm	4	See “Plastic DIN 7985 M3 8 mm”		
DIN 933 M6 16 mm	1	4,71 €	4,71 €	https://www.ebay.de/itm/125956745082?hash=item1d539b677a:g:FGwAAOSwSohkdvvK
DIN 933 M6 20 mm	1	4,84 €	4,84 €	https://www.ebay.de/itm/125956745082?hash=item1d539b677a:g:FGwAAOSwSohkdvvK

NOTE: links should be used as an example where to buy the parts, and may not be active in the future (depending on the stock availability).

## 5 Build instructions

**Fig. 7:**
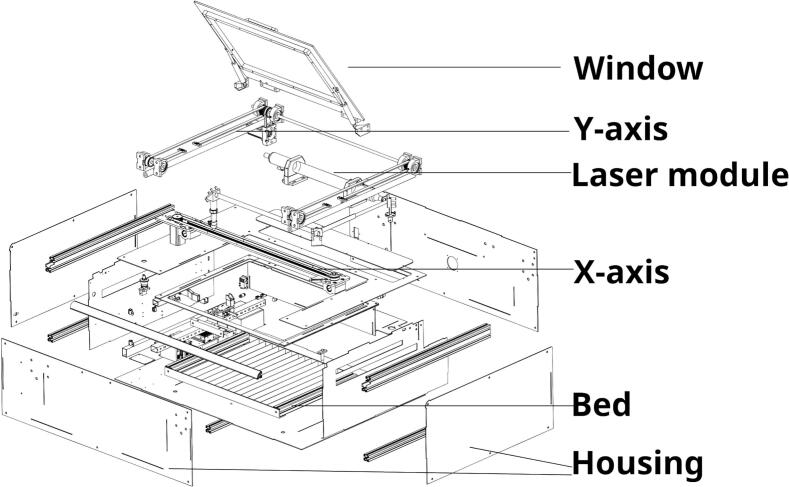
Simplified exploded view of OLSK Small Laser V2: A global view of the machine’s mechanical parts, further elaborated in the more detailed Assembly Workbook. Exploded view is based entirely on the STEP design file in “CAD OLSK Small Laser V2”.

The OLSK Small Laser V2 is an elaborate machine. Fig. 7, above illustrates a simplified exploded view of the different components in the design, excluding the electronic components. The machine design is inspired by previous iterations (i.e. Fabulaser Mini and OLSK Small Laser V1). Due to the complexity and number of parts and sub-assemblies, the assembly manual for OLSK Small Laser V2 is still under development; the assembly steps are therefore here presented as a workbook (Table 5), which divides the assembly in logical steps, and is the base of the web-based assembly manual described earlier. For the Electrical and electronics part, the Wiring Schematic can be followed (Fig. 10).

**Table 5:** OLSK Small Laser V2 Workbook.

**Step**	**Step Title**	**Parts list**		**Tools list**		**Remarks**
		**Qty**	**Part name**	**Qty**	**Tool name**	
**1**	Bottom Panel	1	Bottom Panel Aluminum 3 mm	1	Allen Key 5.0	Do not tighten the screws yet for further alignment.
		6	ISO 7380 M8 10 mm	1	Allen Key 4.0	On the right profile, insert Nuts 6 M5 and attach it to the Bottom Panel Aluminum 3 mm, with the Cable Duct 25x40 700 mm on top.
		3	ISO 7380 M5 12 mm	1	Ruler	Position the Cable Duct 25x40 700 mm starting about 45 mm from the front of the machine.
		3	Top Aluminum Profile 30x60 Nut 8 800 mm			
		1	Cable Duct 25x40 700 mm			
		8	Nut 8 M8			
		4	Nut 6 M5			
**2**	Attach Electronics	2	Stepper Motor Driver DM556T	1	Allen Key 2.5	All Stepper Motor Driver DM556T should have the terminals pointing outwards.
		1	Laser Power Supply MYJG50W			
		1	Controller PCB			
		4	Plastic DIN 7985 M3 8 mm			
		4	Standoff Male Female M3 20 mm			
		8	ISO 7380 M4 10 mm			
**3.1**	Inner Right Panel	1	Inner Panel Right Aluminum 3 mm	1	Allen Key 3.0	Attach the parts before inserting the panel in the Bottom Panel Aluminum 3 mm.
		1	Logic Power Supply LRS-100–24			The Cable Glands should be turned to the inside
		1	Motor Power Supply RSP-500-48A			
		3	DIN 912 M3 6 mm			
		10	ISO 7380 M4 10 mm			
		2	M12x1.5 Cable gland			
		6	Wire Fixers			
**3.2**	Y-motor	1	Cable Duct 25x40 700 mm	1	Allen Key 3.0	Fix the Y Pulley HTD 5 M 16 T 9 mm on the motor shaft aligning it to the edge, with the setscrews towards the outer side.
		5	DIN 912 M4 10 mm	1	Wrench 5.5	Tighten the set screws of the Y Pulley HTD 5 M 16 T 9 mm to the flat part of the motor shaft.
		4	DIN 985 M5			Insert the belt before fixing the motor on Y Motor Holder 3D Printed With Inserts M8.
		1	Y Motor Belt HTD 5 M 9 mm 305 T			Fix the Cable Duct 25x40 700 mm after attaching the motor. They should not be touching each other.
		1	Y Pulley HTD 5 M 16 T 9 mm			The cables of the motor should be pointing inside or down. Really tighten the setscrews of the Y Pulley HTD 5 M 16 T 9 mm. With loctite if possible
		1	Stepper Motor 24HS39-4204D with Cable 4x0.5 mm 100 cm ground shield			
		1	Y Motor Holder 3D Printed With Inserts M8			
		2	ISO 7380 M8 16 mm			
		4	DIN 912 M5 30 mm			
**4**	Inner Left Panel and Middle Panel	1	Inner Panel Left Aluminum 3 mm	1	Allen Key 5.0	The side hole of the Separator Panel Aluminum 3 mm needs to point to the left of the machine (from the front view).
		1	Separator Panel Aluminum 3 mm			
		2	Window Connector 20x20 3D Printed			
		4	ISO 7380 M8 30 mm			
		4	DIN 985 M8			
**5**	Laser Panel	1	Laser Panel Aluminum 3 mm	1	Allen Key 3.0	The base of the First Mirror Holder 25 mm should be fixed upside-down on the bottom of the Laser Panel Aluminum 3 mm.
		1	First Mirror Holder 25 mm	1	Allen Key 4.0	Use Loctite to fix the rod of the First Mirror Holder 25 mm
		2	Laser Tube Holder 45–80 mm			
		8	DIN 912 M5 16 mm			
		2	ISO 7380 M4 10 mm			
**6**	Pistons	2	Piston 100 N Length 250 Travel 80 mm	1	Wrench 13	The Piston 100 N Length 250 Travel 80 mm rod should point up.
		2	Piston Support Right and Left 3D Printed	1	Allen Key 5.0	Insert the DIN 125 M8 between the screw and the piston.
		8	DIN 985 M8			
		2	DIN 125 M8			
		4	ISO 7380 M8 20 mm			
		2	ISO 7380 M8 55 mm			
**7**	Sensor Holders	1	Window Sensor PS-3150 with cable 2x0.25 160 cm ground shield	1	Allen Key 2.5	
		1	Window Sensor Holder 3D Printed	1	Wrench 5.5	
		4	DIN 912 M3 12 mm			
		2	DIN 912 M3 8 mm			
		4	DIN 985 M3			
		1	Y Endstop Holder 3D Printed			
		1	Inductive Probe LJ12A3-4-Z/AY with cable 3x0.25 mm 140 cm ground shield			
**8**	Back Panel	1	Back Panel Aluminum 4 mm	1	Allen Key 3.0	To fit the Back Panel Aluminum 4 mm, move the machine slightly out of the table. Use a rubber hammer if necessary.
		2	Water Connector 9–9 mm Screw 11.7 mm	1	Allen Key 5.0	Fix the Air Connector Press Fit 6–6 mm in the left hole and the Chiller Connector 3 Pole with 20 cm 2x0.25 mm cable no shield in the right hole.
		1	Air Connector Press Fit 6–6 mm	1	Wrench 13	The threads of the Water Connector 9–9 mm Screw 11.7 mm should be inside
		1	Chiller Connector 3 Pole with 20 cm 2x0.25 mm cable no shield	1	Rubber Hammer	
		1	Power Plug 48x28x27mm M4	1	Wrench 19	
		1	Fuse 6.3A 5x20mm			
		6	ISO 7380 M4 10 mm			
		1	Exhaust Adapter 3D Printed			
		5	ISO 7380 M8 10 mm			
**9.1**	Bed Frame	2	Bed Bracket 40x40x3 700 mm	1	Allen Key 5.0	
		2	Aluminum Profile 40x40 Nut 8 500 mm			
		4	ISO 7380 M8 20 mm			
**9.2**	Bed Legs	4	DIN 912 M8 130 mm	1	Wrench 13	
		4	DIN 985 M8			
**9.3**	Bed Lamella	15	Bed Lamella Aluminum 35x40x0.8 500 mm	1	Paper Cutter or Scissors	The Bed Lamella Aluminum 35x40x0.8 500 mm should be placed with the larger side down.
		1	Tesa Double Side Tape 50 mm 5 *m*			For the Bed Lamella Aluminum 35x40x0.8 500 mm, place it pointing backwards if needed.
**9.4**	Inserting the Bed	4	DIN 9021 M8 Washer			
**10**	Front Panel	1	Front Panel Aluminum 4 mm	1	Allen Key 5.0	After fixing the Front Panel Aluminum 4 mm, tighten all the Bottom Panel Aluminum 3 mm screws.
		6	Nut 8 M8			Insert the Nut 8 M8 in the outer side of the profiles, 3 units in each.
		3	ISO 7380 M8 10 mm			
		2	Pipe Holder M6 10 mm			
		2	ISO 7380 M6 16 mm			
		2	DIN 985 M6			
**11**	Y-axis Guide	2	Y Shaft 25 mm 800 mm Tapped M12	1	Allen Key 6.0	Insert theY Linear Bearing SCE25UU in the guide before fixing it.
		1	Y Linear Bearing SCE25UU Left	1	Wrench 10	Work with 2 people.
		1	Y Linear Bearing SCE25UU Right			Insert the DIN 933 M6 12 mm in the Y Linear Bearing SCE25UU Right.
		4	ISO 7380 M12 20 mm			
		1	DIN 933 M6 12 mm			
**12.1**	Y-axis Motion Back 1	4	Pillow Block 12 mm UCPA20	1	Allen Key 6.0	The Pillow Block 12 mm UCPA20 need to be fixed with the setscrews pointing left (right side of the machine looking from the front).
		8	ISO 7380 M10 12 mm			
**12.2**	Y-axis Motion Back 2	1	Shaft 12 mm 1000 mm	1	Lubricant	For inserting the Shaft 12 mm 1000 mm, the bearings of the Pillow Block 12 mm UCPA20 can tilt. Use some force and lubricant.
		2	Y Pulley HTD 5 M 26 T 15 mm	1	Rubber Hammer	Pulleys need to be inserted while inserting the shaft, with setscrews pointing left (right side of the machine looking from the front).
		4	Setscrew (check if inserted in pulleys)			Y Pulley HTD 5 M 26 T 15 mm
		1	Y Pulley HTD 5 M 26 T 9 mm			Start inserting the shaft from the left side of the machine.
**12.3**	Y-axis Motion Front	4	Pillow Block 12 mm UCPA20	1	Allen Key 6.0	
		2	Y Pulley HTD 5 M 26 T 15 mm			
		2	Setscrew (check if inserted in pulleys)			
		1	Shaft 12 mm 100 mm			Left
		1	Shaft 12 mm 150 mm			Right
		8	ISO 7380 M10 12 mm			
**13.1**	Wiring DC	1	Controller Power Cable 2x1mm 70 cm ground shield	1	Screwdriver slotted small	Connect the Window Sensor PS-3150 with cable 2x0.25 160 cm ground shield, Chiller Connector 3 Pole with 20 cm 2x0.25 mm cable no shield and Inductive Probe LJ12A3-4-Z/AY with cable 3x0.25 mm 140 cm ground shield
		2	Driver Power Cable 2x1mm 60 cm ground shield	1	Screwdriver Phillips	
		1	Laser Logic Cable 3x0.25 90 cm ground shield			
		2	Driver Logic Cable 4x0.5 mm 40 cm ground shield			
		1	Resistor 1 K			
		2	AC Jumper 1x1.5 mm 8 cm			
		2	Wago Connector 2 slots			
		1	Wago Connector 5 slots			
**13.2**	Wiring AC	5	Wago Connector 3 slots	1	Screwdriver Phillips	
		1	Wago Connector 2 slots			
		1	Wago Connector 5 slots			
		1	Emergency Button Cable 2x1.5 mm 110 cm			
		1	AC Power Cable 3x1.5 mm 20 cm			Laser Power Supply Cable
		1	AC Power Cable 3x1.5 mm 20 cm			Logic Power Supply Cable
		1	AC Power Cable 3x1.5 mm 20 cm			Motor Power Supply Cable
		1	AC Extension Cable 3x1.5 mm 25 cm			
		1	Earth Cable AC 1x1.5 mm 15 cm			
**14.1**	Preparing the X-axis 1	1	X Plate Aluminum 8 mm	1	Allen Key 4.0	Push the X Linear Guide HGR15 T 850 mm to the outside to align and tighten the screws all at the same time.
		1	X Linear Guide HGR15 T 850 mm			
		13	DIN 912 M5 12 mm			
**14.2**	Preparing the X-axis 2	2	DIN 912 M3 8 mm	1	Allen Key 2.5	Hammer the X Shaft 12 mm 35 mm inside the X Plate Aluminum 8 mm.
		1	X Shaft 12 mm 35 mm	1	Allen Key 5.0	Fix the X Endstop Holder POM 6 mm in the correct position.
		1	X Left Pulley HTD 5 M 28 T 9 mm	1	Hammer	Do not tighten the Y Belt Attacher Aluminum 6 mm attachers yet.
		2	Setscrew (check if inserted in pulleys)	1	Lubricant	
		4	Y Belt Attacher Aluminum 6 mm			
		8	DIN 912 M6 16 mm			
		1	X Endstop Holder POM 6 mm			
		1	Inductive Probe LJ12A3-4-Z/AY with cable 3x0.25 mm 320 cm			
**14.3**	Preparing the X-axis 3	1	Second Mirror Holder 25 mm	1	Allen Key 4.0	Insert the X Carriage QHH15CA1T1200Z0C carefully while removing the plastic one.
		1	Laser Mirror Spacer 3D Printed			
		2	DIN 912 M4 16 mm			
		1	X Carriage QHH15CA1T1200Z0C			
**15**	Attaching the X-axis	1	Cable Ties min. 150 mm 100 pieces	1	Allen Key 5.0	Pass the X-axis through the bottom slot of the Inner Panel Left Aluminum 3 mm.
		8	ISO 7380 M8 20 mm			Tighten the cable of the Inductive Probe LJ12A3-4-Z/AY with cable 3x0.25 mm 320 cm on the body of the sensor
**16**	Attaching the X-motor	1	Stepper Motor 24HS39-4204D with Cable 4x0.5 mm 150 cm ground shield	1	Allen Key 3.0	The cable of the motor should point outwards
		4	ISO 7380 M5 25 mm	1	Wrench 8	
		4	DIN 985 M5			
		1	X Right Pulley HTD 5 M 28 T 9 mm			
		2	Setscrew (check if inserted in pulleys)			
**17**	Install LEDs	2	Led Strip 24 V Warm White 40 cm with cable 2x0.75 70 cm + 2x0.75 230 cm	1	Allen Key 3.0	The cables of the Led Strip 24 V Warm White 40 cm should point to the holes in the Inner side panels.
		2	Cable Ties min. 150 mm 100 pieces			Pass the left Led Strip 24 V Warm White 40 cm cable through the holes on the Middle and Laser Panel
**18**	Top Profiles	2	Top Aluminum Profile 30x60 Nut 8 800 mm	1	Allen Key 6.0	Insert the Nut 8 M8 in the outer side of the profiles, 3 units in each.
		6	ISO 7380 M8 10 mm			
**19**	Y-belts	2	Y Belt HTD 5 M 15 mm 2000 mm	1	Large Pliers	To tighten the Y Belt HTD 5 M 15 mm 2000 mm, loosen the front Pillow Block 12 mm UCPA201 first. Then fix one end of the belt with the Y Belt Attacher Aluminum 6 mm gripping the belt teeth. Pass the belt around the Y Pulley HTD 5 M 26 T 15 mm and fix the other end of the belt. Then tighten the Pillow Block 12 mm UCPA201
**20**	Install Laser Head	1	Head Holder Aluminum 6 mm	1	Allen Key 2.5	The side hole of Laser Head Mirror 25 Lens 20 should point to the second mirror
		1	Laser Head Mirror 25 Lens 20			
		4	DIN 912 M4 12 mm			
		4	DIN 912 M4 10 mm			
**21**	Install X-chain	1	Air pipe 4x6mm 5 *m*			Pass the Air pipe 4x6mm 5 m through the Cable Chain inner size 15x15 outer size 24x20 2000 mm before fixing it
		1	Cable Chain inner size 15x15 outer size 24x20 2000 mm			Fix the Cable Chain inner size 15x15 outer size 24x20 2000 mm in the right position. If needed, invert the ending piece.
		3	DIN 912 M4 35 mm			Fix the chain to the X-axis with cable ties, together with the Inductive Probe LJ12A3-4-Z/AY with cable 3x0.25 mm 320 cm. Position it under the X-axis.
		1	X Chain Spacer 3D Printed			
		3	Cable Ties min. 150 mm 100 pieces			
		1	Y Cable Organizer 3D Printed			
		3	DIN 912 M4 12 mm			
		2	DIN 912 M3 8 mm			
**22**	Align X-axis	2	Template	2	Template or two identical objects	Align the X-axis using the templates as reference to set an equal distance from the Front Panel Aluminum 4 mm. Then tighten all the pulley of the Y-axis.
**23**	Insert X-belt	1	X Belt HTD 5 M 10 2075 mm	1	Allen Key 3.0	After inserting theX Belt HTD 5 M 10 2075 mm around the X Right and Left Pulleys HTD 5 M 28 T 9 mm, tighten it by unscrewing the DIN 933 M6 12 in the Y Linear Bearing SCE25UU Right.
		1	Belt Attacher Aluminum 6 mm			
		2	DIN 912 M4 16 mm			
		2	Washer M4			
**24**	Installing the Y-chain	1	Cable Chain inner size 25x18 outer size 35x23 1000 mm	1	Allen Key 3.0	Make sure the Cable Chain inner size is 25x18 mm & outer size 35x23 mm with length being 1000 mm..
		6	DIN 912 M5 12 mm			Insert theAir pipe 4x6mm 5 m, the Probe LJ12A3-4-Z/AY with cable 3x0.25 mm 320 cm cable and the Stepper Motor 24HS39-4204D with Cable 4x0.5 mm 150 cm ground shield cables in the Cable Chain inner size 25x18 outer size 35x23 1000 mm. The segments can be opened with a flat screwdriver.
**25**	Water Pipes	1	Water Pipe Inside Silicon 1 m 7x10mm			
		1	Water Pipe Inside Silicon 1 m 7x10mm			
		2	Water Pipe Outside inside diameter 9 mm 2 m length			
		2	Cable Ties min. 150 mm 100 pieces			
**26**	Laser Tube	1	Laser Tube CO2 40 W 50x720mm	1	Cutter	Remove the top part of theLaser Tube Holder 45–80 mm to insert the Laser Tube CO2 40 W 50x720mm.
		2	Cable Ties min. 150 mm 100 pieces			Connect the Water Pipe Inside Silicon 1 m 7x10mm with the Laser Tube CO2 40 W 50x720mm. Fix the connections with Cable Ties.
**27.1**	Wiring DC 2	2	Laser connector (screw terminal mammut) 2 pos	1	Screwdriver slotted small	Connect the motors,Probe LJ12A3-4-Z/AY with cable 3x0.25 mm 320 cm and Led Strip 24 V Warm White 40 cm with cable 2x0.75 70 cm + 2x0.75 230 cm
		1	Laser (−) Cable			Connect the Laser (+) and Laser (−) Cable and cover the terminals with a shrinking tube. Make sure the laser attachment is well fixed.
**27.2**	Cable Management		Cable Tie			
**28.1**	Sub and Spacer Sides Panels	1	Sub Window Right Aluminum 3 mm			
		1	Sub Window Left Aluminum 3 mm			
		1	Window Spacer Right Aluminum 3 mm			
		1	Window Spacer Left Aluminum 3 mm			
**28.2**	Top Panel	1	Top Panel Aluminum 3 mm	1	Screwdriver Phillips	Insert the Nut 8 M8 before placing the Top Panel Aluminum 3 mm.
		6	ISO 7380 M8 10 mm			Connect theEmergency Button Cable 2x1.5 mm 110 cm to the Emergency Button With Key AS22-B142.
		6	Nut 8 M8			
		1	Emergency Button With Key AS22-B142			
**28.3**	Sub and Spacer Back Panels	1	Sub Window Back Aluminum 3 mm	1	Allen Key 5.0	Fit the Sub Window Back Aluminum 3 mm and the Window Spacer Back Aluminum 3 mm under the Top Panel Aluminum 3 mm with the holes aligned.
		1	Window Spacer Back Aluminum 3 mm	1	Wrench 13	
**28.4**	Subpanel Front	1	Sub Window Front Aluminum 3 mm	1	Allen Key 5.0	
		6	ISO 7380 M8 20 mm	1	Wrench 13	
		6	DIN 985 M8			
**29.1**	Prepare the Fillet Profile	1	Fillet Aluminum Profile 30x30 Nut 8 1145 mm	1	Wrench 13	Fix the Front Support 3D Printed and theFront Bracket 30x20x3 200 mm to the Fillet Aluminum Profile 30x30 Nut 8 1145 mm before fixing it to the Front Panel Aluminum 4 mm.
		1	Front Support 3D Printed			
		2	DIN 912 M4 14 mm			
		2	Washer M4			
		2	Nut 6 M4			
**29.2**	Attach Fillet Profile	2	ISO 7380 M8 20 mm			Do not tighten the ISO 7380 M8 20 mm yet for further alignment.
		1	Front Bracket 30x20x3 200 mm	1	Allen Key 5.0	
		2	Nut 8 M8			
		4	ISO 7380 M8 10 mm			
		2	DIN 985 M8			
**30.1**	Prepare Window 1	1	Laser Window Acrylic 750x600 6 mm	1	Allen Key 4.0	Laser Window Acrylic 750x600 6 mm holes are not symmetric! The profiles holes should be matched before inserting the corner connectors
		1	Window Front Profile Aluminum 20x20 599 mm	1	Wrench 10	
		1	Window Back Profile Aluminum 20x20 599 mm	1		
		1	Window Left Profile Aluminum 20x20 380 mm	1	Allen Key 3.0	
		1	Window Right Profile Aluminum 20x20 380 mm			
		4	Window Angle Press Fit 20x20			
**30.2**	Prepare the Window 2	9	ISO 7380 M6 35 mm	1	Allen Key 2.5	
		9	DIN 985 M6			
		2	DIN 912 M4 35 mm			
		1	Window Handler 192 mm			
		1	Window Magnet PS-3150			
		2	DIN 912 M3 6 mm			
		2	Hinge GN 237-ZD-60–60-A-SW			
		4	DIN 7991 M8 25 mm			
		4	DIN 985 M8			
**31**	Install Window	1	Prepared Window	1	Allen Key 5.0	Insert theDIN 125 M8 between the piston and the DIN 985
		4	DIN 7991 M8 50 mm	1	Allen Key 6.0	Do not touch the surface of the mirrors.
		2	Standoff M8 15–8 Male-Female	1	Wrench 13	
		2	ISO 7380 M8 30 mm			
		2	DIN 125 M8			
		6	DIN 985 M8			
**32**	Insert Mirrors	3	Laser Mirror 25 mm			
**33**	Prepare for Calibration	1	Air pump Hailea ACO-318	1	Digital Level meter	Semi-tighten the screws of the Laser Tube Holder 45–80 mm and First Mirror Holder 25 mm.
		1	Water Chiller CW-3000	1	Caliper	Test the water.
		1	5L Distilled Water			Align the Laser Tube Holder 45–80 mm with the caliper
		2	Water Pipe Outside inside diameter 9 mm 2 m length			Make sure the Laser Tube CO2 40 W 50x720mm is parallel to the frame and horizontally leveled.
		2	Clamps (included in Water Chiller CW-3000)			Pre-align visually First Mirror Holder 25 mm, Second Mirror Holder 25 mm and Laser Tube CO2 40 W 50x720mm.
		1	Chiller Cable 2 cores min. outer diameter 6.5 mm 2 m with 2 x chiller connectors			
		1	Air pipe 4x6mm 5 *m*			
		1	Air pipe adapter 6–10 mm with pipe 10 mm			
**34**	Laser Calibration	1	Power Cord (computer style) at least 2 *m*			Always use 2 layers tape on the mirror, and one layer to align
		1	Masking tape			
**35**	Insert Lens	1	Laser Lens 20 mm 50.8 mm			Place it with the flat surface down.
		2	Rubber Rings 20 mm			Do not touch the surface of the lens.
		1	Hot Glue			
**36**	Fix Top Back Panel	1	Cover Panel Aluminum 3 mm	1	Allen Key 2.5	
		6	ISO 7380 M4 10 mm			
**37**	Insert Side Panels	1	Left Panel Aluminum 3 mm	1	Allen Key 5.0	Tighten the screws of theFront Bracket 30x20x3 200 mm and theFront Support 3D Printed.
		1	Right Panel Aluminum 3 mm			To fix the Right and Left Panels Aluminum 3 mm, insert them in the slot of the Front Panel Aluminum 4 mm first.
		14	ISO 7380 M8 10 mm			
		1	USB Cable Inside			
		2	Plastic DIN 7985 M3 8 mm			
**38**	Install Air Filter / Radial Fan	1	Air Filter Airmex AM03 or similar / Radial Fan 150 mm 150 W Neverest RV-B			
		1 or 2	Exhaust hose 80 mm 1.5 *m*			
		2 or 3	Hose Clamps 80 mm			
		1	Foot Switch			

The table below is the current workbook, and reports the main steps for the assembly of OLSK Small Laser V2.

**Fig. 8:**
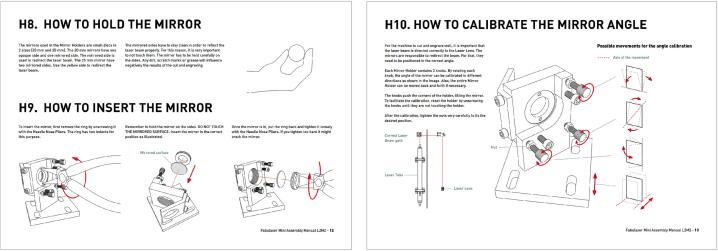
Information to calibrate.

**Fig. 9:**
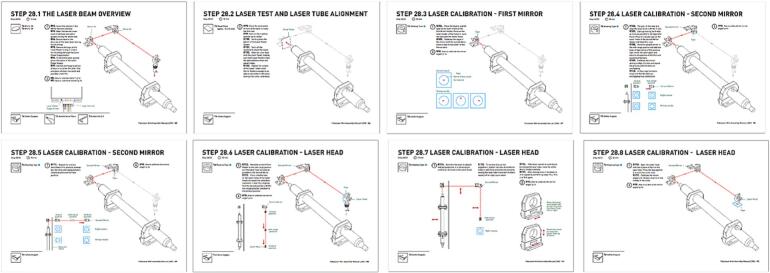
Steps to calibrate.

Step 34 (Calibration) is the most delicate and sensitive assembly step. It may require great trial and error and the experience is a key factor for speeding this step. See (Fig. 8, Fig. 9).

In the manual of Fabulaser Mini V2 (pages 12 and 13; pages 85 to 92) [13] are contained the following information:

● How to hold the mirror − page 12
● How to insert the mirror − page 12
● How to calibrate the mirror angle − page 13
● The laser beam overview − page 85
● Laser test and laser tube alignment − page 86
● Laser calibration − first mirror − page 87
● Laser calibration − second mirror − pages 88 and 89
● Laser calibration − head −pages 90, 91 and 92

Calibration is potentially dangerous due to the shooting of the laser without the safety feature activated. For this reason, it must be carried on carefully and with all the precautions, as reported in the assembly manual of Fabulaser Mini V2 (page 4):

● The laser calibration should be carried out with a minimum of 2 people: one checking the laser path and the other operating the computer
● Only UGS software should be running on the computer; if UGS is running too slow, another computer should be used
● To avoid distractions, phones should not be used and should be in silence mode
● Make sure to do the calibration in a room without anything flammable that could potentially set on fire
● The room where the calibration is carried out should not have other people inside
● Calibration should be performed far away from the windows, or with the windows’ blinds closed
● Other people should be informed about the calibration, to avoid that somebody enters the room suddenly without notice
● All mechanical parts should be checked to have been correctly installed. All the moving parts should be checked that can move freely
● All the wiring should be checked to have been correctly connected and to have been tested beforehand with the electricity power socket (220 V)
● Water connections should be checked to have been firmly done so that the water is flowing (to the proper direction)
● Water Chiller should be checked to be switched on.
● Transparent plastic safety goggles should always be worn
● Long clothes should always be worn
● The operators should be ready to push the emergency button if the laser stays on, or if the beam is going in the wrong direction
● All the Mirror Holders, the Laser Head and the Mirrors should be checked to ensure that they are installed before starting the calibration
● The laser tube should be checked to ensure that it is installed pointing to the correct direction; the end with a hole, through which the laser will come out, and should be pointing to the First Mirror
● The laser tube should be checked to ensure that is not damaged, cracked, or leaking water from any point
● The laser path should be checked to ensure that it is hitting the mirror(s); if not sure, something to block the laser beam from hitting anything should be used on the first test. It is recommended to use a flat wood plank.
● The operator should never stand in the direction of the laser beam.
● Only the necessary housing parts should be removed. The rest should be kept attached as a protection
● The machine window should always be closed to activate the window switches
● Tape should not be sticked to the mirror, but only to the holders
● Masking tape which is thick enough should be used. A single test should be performed to make sure it does not set on fire. If it is too thin, it is possible to use two overlapping pieces of tape
● A fire extinguisher or a bucket of water should be kept close to the machine

**Fig. 10:**
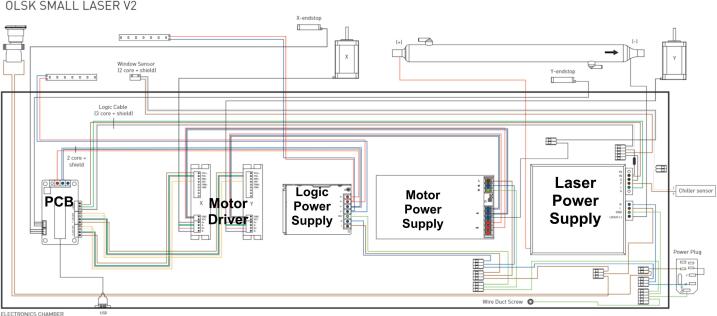
Wiring Schematics.

The Wiring Schematics in Fig. 10 shows the connections between the components of the electric/electronics components.

The wiring schematic diagram illustrates the core components of the OLSK Small Laser V2. The Laser tube is where the laser beam is generated and it is connected to the Laser Power Supply, which provides the voltage and current to excite the gas inside the tube, creating a beam. The Laser power supply energizes the laser tube and there are control signal connections running from it to the main PCB. The Motor Power Supply provides power to the stepper motors that move the laser assembly along the X and Y axes, allowing the laser beam to be precisely positioned for cutting or engraving workpieces. The Motor Drivers are connected to the Logic Power Supply and the Motor Power Supply, they receive control signals from the logic board, and help move the laser head to the desired position. The Logic Power Supply provides power to the logic circuits, which include the necessary electronics for the operation of the laser cutter. The PCB, enables the laser cutter and the computer to communicate, where data is interpreted to control the movement of the laser head. The Endstops (X and Y) are inductive probes that the moving part of the machine might activate, to let the control system know when a limit has been reached (preventing unwanted crashes, common in old proprietary laser cutter models). Similarly, the Window Sensor also monitors the opening and closing of the machine. The Chiller sensor uses water to keep the laser tube cool. The chiller sensor monitors the temperature and ensures that the laser tube does not overheat. LED strips are used for the illumination inside the machine, so that the user can see the workpiece and the laser head's path. Additionally, wire duct screws allow for the management and tidiness of the machine and the electronic chamber depicts the separation of the Electronics from where the laser cutting or engraving operations happen.

**Fig. 11:**
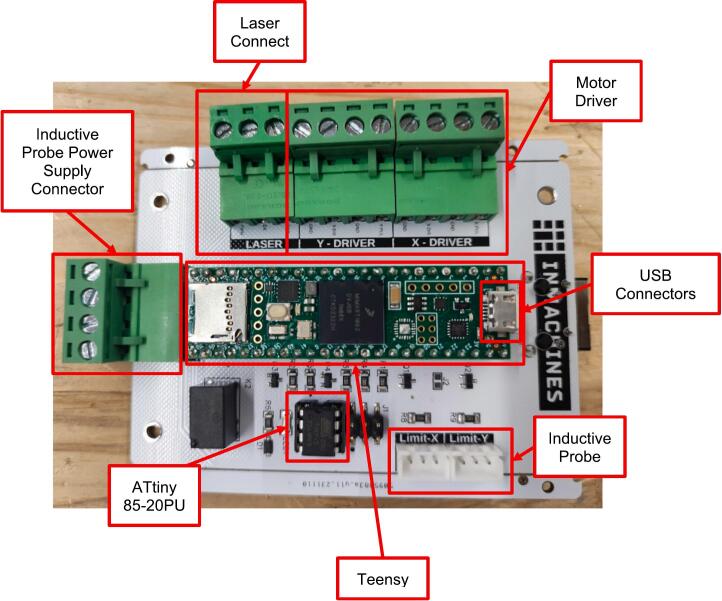
Controller PCB and its components.

The laser cutter is driven by the Laser Controller PCB V1.7 (Fig. 11).

The Controller PCB is composed of:

● A shield on which all the components are placed
● ATTiny 85-20PU/ATTiny 85-20PU
● Teensy 4.1
● Connectors
  ○ Inductive Probes
  ○ Motor Drivers
  ○ USB
  ○ Laser
  ○ Inductive Probes Power Supply

The function of the Laser Controller PCB is to enable the communication between the laser cutter and the computer, and to drive motors and laser source to perform laser cutting and engraving jobs.

A microcontroller is an embedded system similar to a simple computer with everything integrated, which works with systems, such as sensors and actuators, in real time. The used microcontroller is the iMXRT1062 integrated in the Teensy 4.1, which is composed of a chip with Cortex ARM M7 architecture and has inside everything needed to execute the machine code (RAM memory, EPROM, Flash memory, USB…). Since it has a BGA package, the chip is hard to embed, therefore the Teensy 4.1. The Teensy is integrated in the laser cutter thanks to a customized shield (Laser Controller PCB), connected to the Teensy via pins. The Teensy is a generic board with minimum I/O, while for the OLSK Small Laser needs other connectors different from the pin headers and signal transformations. The shield provides:

● Connections for the motor drivers signal conversion and connections for the inductive probes (limit switches) which work at 24 V
● Connections for the laser power supply, where the signal is logically converted from 3.3 V to 5 V
● Power connections for 24 V

The ATtiny is a very simple controller which manages the connection with the laser power supply. When the controller is powered, the teensy needs time to initialize. During the initialization, the Teensy outputs are not stable, therefore the laser may be wrongly activated during the starting phase of the Teensy. To prevent this the ATtiny has a timer that makes the connection from the output of teensy to the laser power supply activated with 6 s delay from the turning on of the controller. The PCB supports ethernet via the Teensy, but in the firmware the ethernet connection is not very stable yet, so the USB connection is to be preferred. Teensy works at 3.3 V, provided by an integrated stepdown voltage regulator from 5 V given by the USB connection.

**Flashing the Teensy 4.1 and the ATtiny 45**

Instructions on how to flash the Teensy 4.1 and the ATtiny 45 may be found in “Electronics OLSK Small Laser V2”.

## 6 Operation instructions

Operation Instructions may be found in “Additional Documentation OLSK Small Laser V2”.

It is very important to take all the necessary precautions:

● The laser cutter should never be left unattended while working
● If needed, the job can be paused (the job can be resumed) or stopped (the job cannot be resumed)
● In case of an emergency (like fire), the emergency button should be pushed immediately: the machine will stop working promptly. The job cannot be resumed

The OLSK Small Laser V2 uses a laser beam and therefore all the precautions must be taken: no skin and eye exposure to the laser beam and attention towards possible fires.

The OLSK Small Laser V2 uses high voltage current and therefore, any access to the electrical and electronic parts and the laser tube must be performed with the machine turned off and unplugged.

## 7 Validation and characterization

**Fig. 12:**
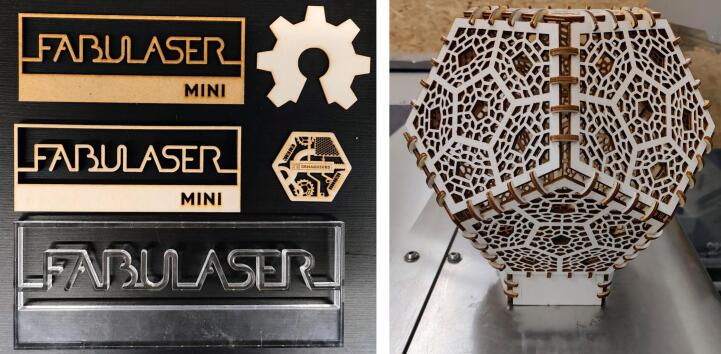
Laser cut work pieces.

OLSK Small Laser V2 has been used to cut cardboard, plywood, acrylic and MDF (Fig. 12).

The OLSK Small Laser system incorporates a comprehensive suite of safety features designed to ensure secure operation. These features include:

● Magnetic Window Sensors: These sensors prevent laser activation when the access window is not securely closed, thereby mitigating the risk of accidental exposure to the laser
● Electrical System Grounding: In order to reduce the risk of electrical shock and help protect the electronic components from power surges
● High Voltage/Low Voltage Separation: The system's design separates high-voltage and low-voltage circuits (AC and DC), minimizing the potential for electrical interference
● Non-Flammable Construction: The extensive use of aluminum in the construction of the laser system reduces the risk of fire
● Isolated Electronics Chamber: The electronics are housed in a separate chamber to provide easy access and protect other parts from failures
● Emergency Stop Mechanism: An emergency button equipped with a key provides a rapid means to cease all laser operations in the event of an emergency
● Inductive Probes as End-Stops: Inductive probes detect the physical limits of axis movement, ensuring that motion ceases before the mechanical boundaries are breached, thus preventing damage to the machinery
● Water Chiller Sensor: The inclusion of a water chiller sensor allows for continuous monitoring of the cooling system's performance, which is essential for maintaining the laser's operational integrity and preventing overheating

**Fig. 13:**
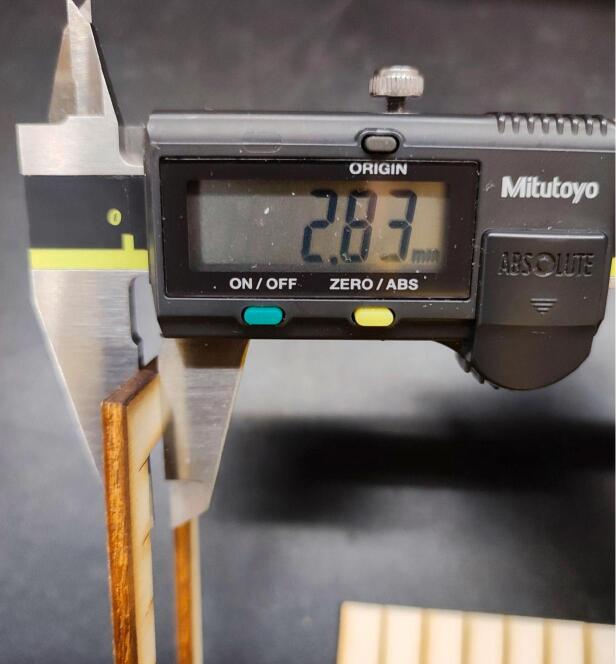
Thickness of the tested plywood.

To validate the reliability of the OLSK Small Laser, the parameters for marking, cutting and engraving were tested for poplar plywood (about 3 mm thickness, as shown in (Fig. 13).

For the measurements, the Mitutoyo Digimatic Caliper (model CD-15APX) was used.

The maximum speed for the OLSK Small Laser V2 is 1000 mm/s, which corresponds to a speed value of 100 (%).

**Fig. 14:**
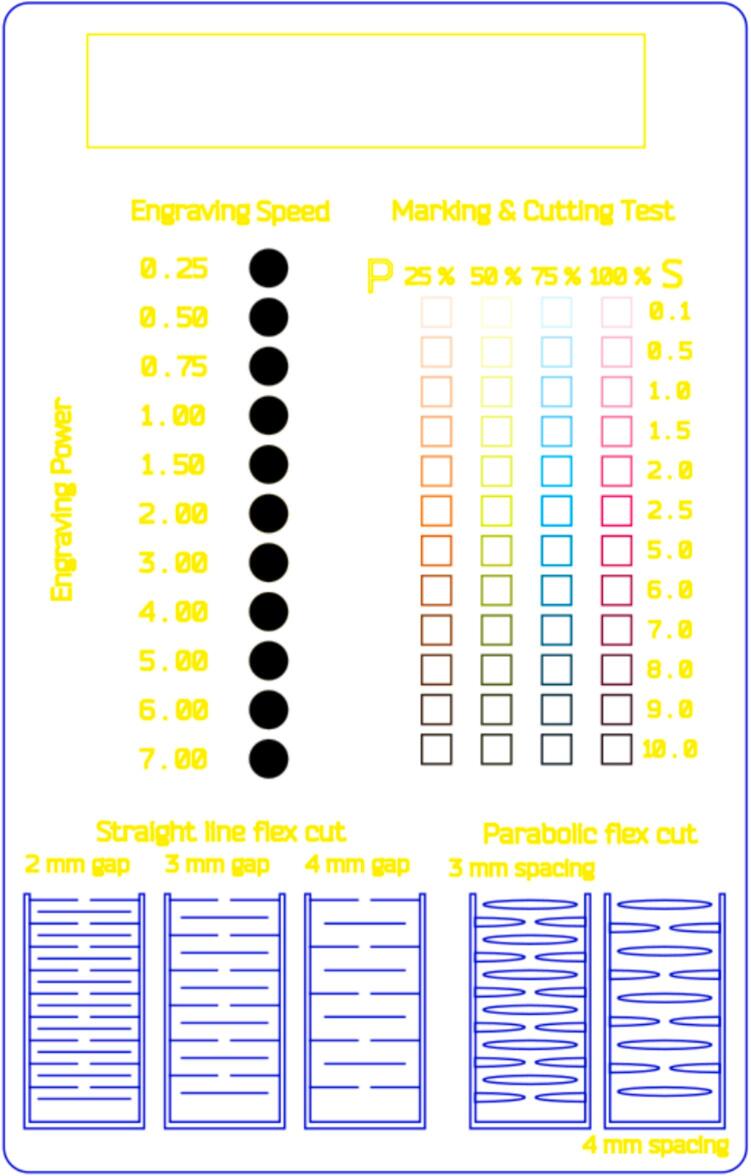
Test Template: SVG file; design based on Mike Murray’s template [14].

A template, shown in Fig. 14, was designed in Inkscape.

**Fig. 15:**
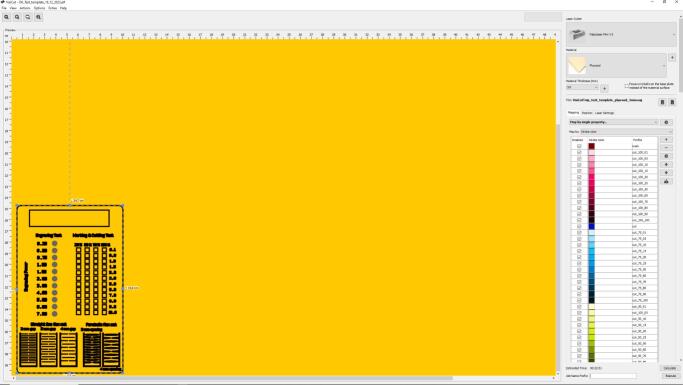
Test Template: Visicut file.

The Gcode was prepared using Visicut, as can be seen in Fig. 15.

### 7.1 Engraving

**Fig. 16:**
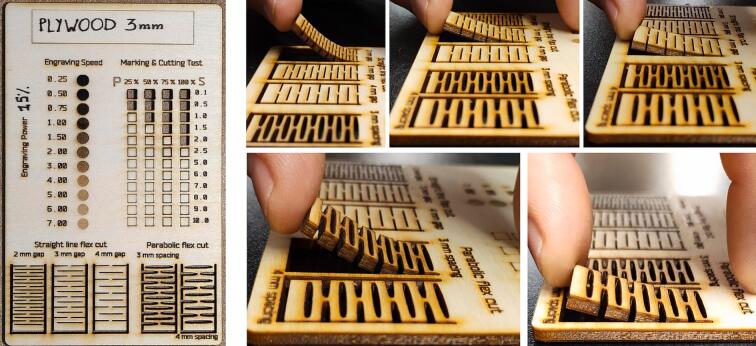
Laser cut test on the left. Living hinges test on the right.

Using an engraving power of 15 %, the speed was varied between 0.25 % (2.5 mm/s) and 100 % (1000 mm/s). The dithering algorithm used was Floyd-Steinberg. See (Fig. 16).

### 7.2 Marking and cutting

A combination of 4 different power values (25 %, 50 %, 75 % and 100 %) and speed values (ranging from 0.1 % to 10 %) were tested.

### 7.3 Living hinges

5 different patterns were tested for living hinges

● Straight line
  ○ 2 mm gap
  ○ 3 mm gap
  ○ 4 mm gap
● Parabolic
  ○ 3 mm spacing
  ○ 4 mm spacing

The kerf was tested for plywood 3 mm thick.

**Fig. 17:**
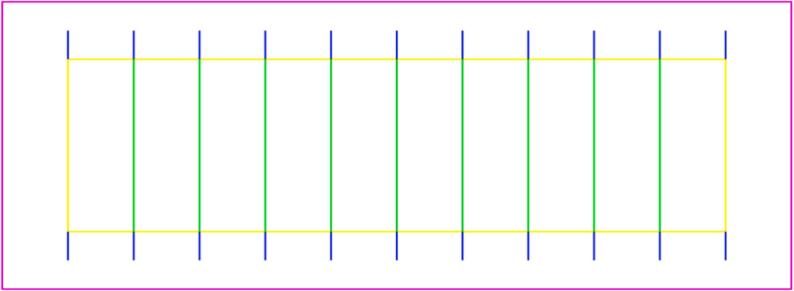
Kerf Test: SVG file.

A simple design, composed of 10 rectangles, was drawn as shown in Fig. 17.

**Fig. 18:**
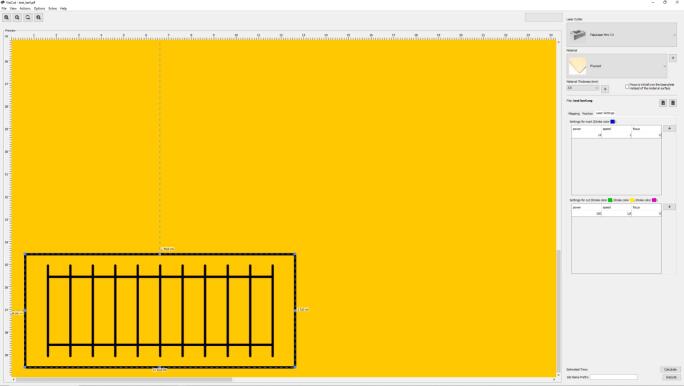
Kerf Test: Visicut file.

The GCode was prepared in Visicut, using the following parameters. See (Fig. 18.

● Green lines, yellow lines and pink lines − cutting
  ○ Speed: 1.8 %
  ○ Power: 100 %
● Blue lines − marking
  ○ Speed: 0.5 %
  ○ Power: 14 %

**Fig. 19:**
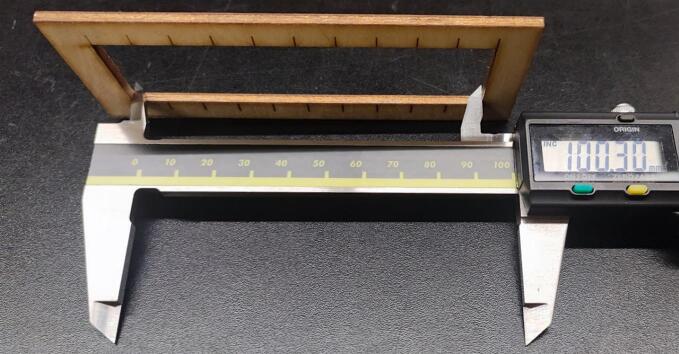
Measurement of the outer rectangle: test 1.

**Fig. 20:**
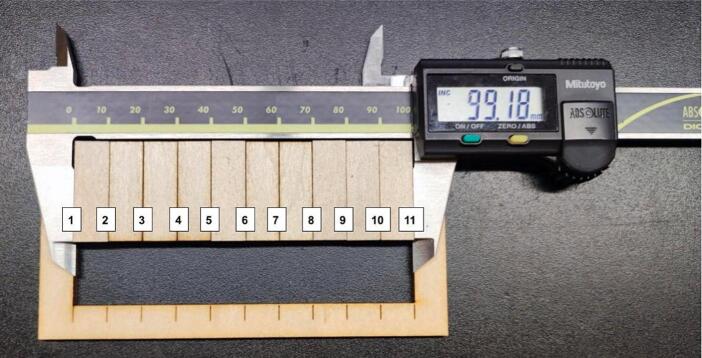
Measurement of the inner rectangles: test 1.

**Fig. 21:**
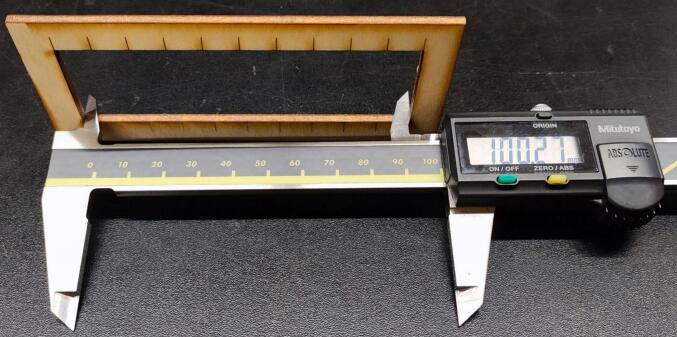
Measurement of the outer rectangle: test 2.

**Fig. 22:**
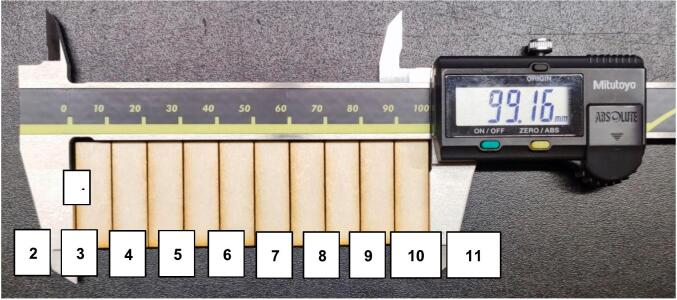
Measurement of the inner rectangles: test 2.

Marking was performed first, followed by the cutting of the green lines, the cutting of the yellow lines and eventually the cutting of the pink lines. The cut parts were measured, as shown in Fig. 19, Fig. 20, Fig. 21, Fig. 22.

The kerf was calculated with the formula:

$$\ker f=\frac{\left(A-B\right)}{C}$$

Two tests were performed:

● Test 1: maximum speed 10000 mm/min

The outside cut measured 100.30 mm, the sum of the inside rectangles measured 99.18 mm.

Where A = 100.29 mm, B = 99.18 and C = 11 (number of cuts).

The calculation resulted in kerf = 0.10 mm.

● Test 2: maximum speed 60000 mm/min

The outside cut measured 100.27 mm, the sum of the inside rectangles measured 99.16 mm.

Where A = 100.27 mm, B = 99.16 and C = 11 (number of cuts).

The calculation resulted in kerf = 0.10 mm.

The OLSK Small Laser V2 showed a consistent kerf at two different maximum speeds.

### 7.4 Relevant use cases

The OLSK Small Laser V2 is used mainly for the production of components and tools of the other machines of the Open Lab Starter Kit or for the replication of the OSLK Small laser V2 itself.

For example, OLSK Small Laser V2 has been used to produce:

• Masks for the precise drilling of holes in OLSK machines components (for example, for the component “Front Bracket 30x20x3 200 mm”)
• Focus tools
• X Endstop Holder POM 6 mm

The decision of using OLSK Small Laser V2 resides in the ideal of self-replicating machines and in the intent to prove this concept. Furthermore, given its reliability, the company InMachines decided to use OLSK Small Laser V2 as its main laser cutter (together with LaserDuo for the production of bigger pieces, such as the window of OLSK Small Laser V2).

**Fig. 23:**
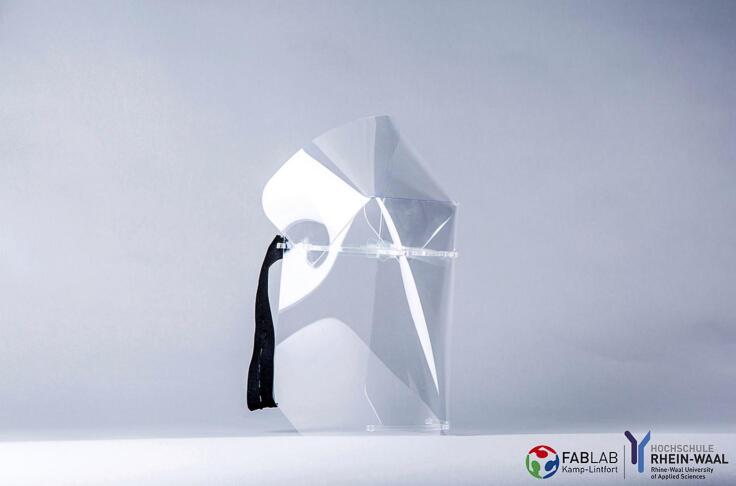
Laser cut face shield: by Christian Spieß, TROK-MEDIA.

Source: https://fablab.hochschule-rhein-waal.de/

**Fig. 24:**
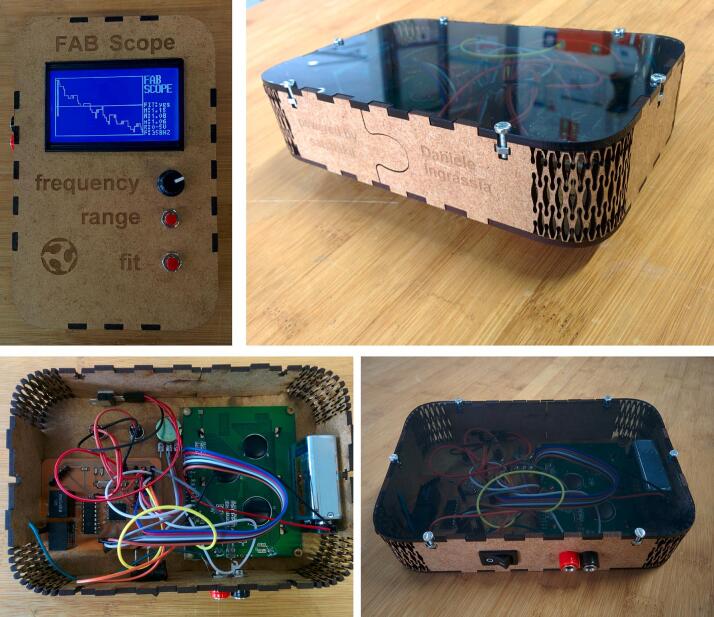
FabScope:

Source: https://github.com/satshas/FabScope.

**Fig. 25:**
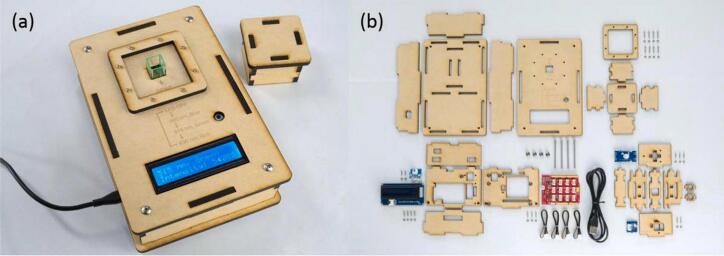
Colorimeter Kuutano [12]. a) Assembled colorimeter. b) The Colotimeter Kuutano Kit.

**Fig. 26:**
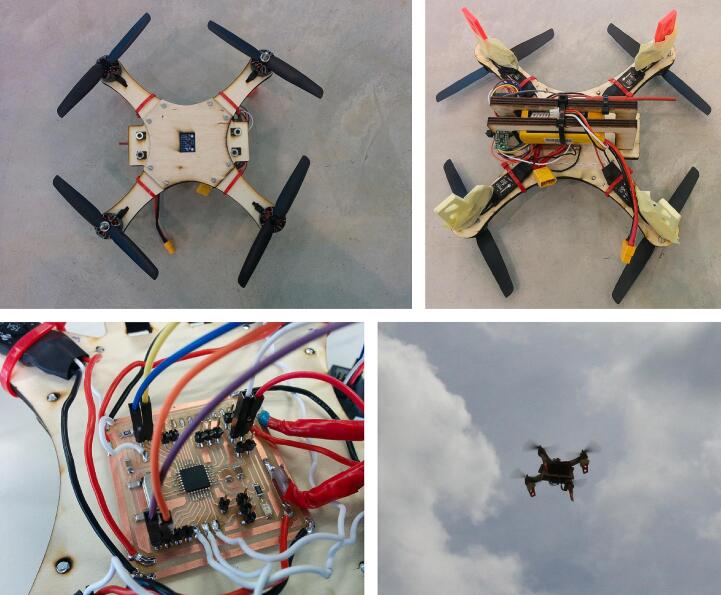
Satshacopter:

Source: https://github.com/satshacopter/satshacopter-250X.

Laser cutters in general have been used in the production of several projects that the authors report as examples of what could be produced with OLSK Small Laser V2. See (Fig. 23, Fig. 24, Fig. 25, Fig. 26).

These projects include:

● Face masks during the COVID pandemic to cut out the polycarbonate face shields

During the COVID 19 pandemic, several makers felt involved in finding solutions to produce protective gears against the spreading of the virus. The Rhine Waal University of Applied Sciences also participated in the effort, with the release of a completely laser cuttable face shield [11].

● FabScope:

FabScope is a publicly available oscilloscope. The case is entirely produced using laser cutting.

The side is laser cut with a living hinge; the pattern made the MDF panel flexible.

● Colorimeter Kuutamo

Colorimeter Kuutamo is an Open Source colorimeter produced entirely using the laser cutting technique.

● Satshacopter:

Satshacopter is a publicly available quadcopter. The frame is entirely made of laser cut plywood.

### 7.5 Future developments

OLSK Small Laser V2 is a prototype and presents its limitations, such as:

● Lower usability compared to commercial machines
● Not being a stand-alone machine (requiring a computer to be operated)
● Needing 2 different programs to run an job
● Need of a chiller
● Still hard laser calibration
● Bigger size and higher weight in comparison of similar commercial laser cutters

The OLSK Small Laser V2, as mentioned above, is not the last iteration of this machine.

In order to produce an Open Source laser cutter with comparable usability to the latest developed commercial machines, these features are planned to be implemented:

● Stand-alone laser cutter: in the OLSK Small Laser V3 will be integrated an Open Source software to manage the machine; the software (OLOS − Open Lab Operating System) is currently under development and will run on a single board computer; UGS and Visicut will not be longer needed
● Camera: a camera to observe the cutting area will be mounted
● Touch screen: a large touch screen will increase usability
● CO_2_ Rf metal laser source: this laser source shows numerous advantages compared to CO_2_ glass pipe laser source:
  ○ It is air cooled and doesn’t require the water chiller
  ○ It uses a lower voltage, making itself safer than CO_2_ glass pipe laser source
  ○ It has a built-in coaxial laser pointer, which makes the calibration safer and easier
● Servo motors instead of stepper motors: a closed loop control is implemented, the servo motors are more reliable and faster; they are smoother in motion and produce less noise
● Tool changer: OLSK Small Laser V3 will implement a tool changer to change laser source and to have a machine that is configurable. Sensors, autofocus and other features and functionalities are also considered for tool changer
● Reduced use of aluminum profiles: t-slotted aluminum profiles are not easily available everywhere and therefore a reduced amount in the next version is aimed
● Z-axis implemented: the Z-axis will be moved up and down for the focus of the laser
● Possibly CE certified

## 8 Conclusion

The OLSK Small Laser V2 has shown to be a reliable machine, which can be produced both industrially and in a Fab Lab or Maker Space. Great attention was given to the safety features, implementing a redundant system of sensors and switches.

Nevertheless, many improvements will be realized in the third design and prototyping iteration (OLSK Small Laser V3), to render this Open Source laser cutter safer, more user friendly and less voluminous.

## CRediT authorship contribution statement

**Daniele Ingrassia:** Writing – review & editing, Writing – original draft, Validation, Supervision, Project administration, Methodology, Conceptualization. **Gaia Di Martino:** Writing – review & editing, Writing – original draft. **J.C. Mariscal-Melgar:** Writing – review & editing, Writing – original draft. **Mohammed Omer:** Writing – review & editing, Writing – original draft. **Liane Sayuri Honda:** Writing – original draft, Conceptualization. **Luisa Lange:** Writing – review & editing, Supervision. **Marc Kohlen:** Methodology, Conceptualization. **Manuel Moritz:** Writing – review & editing, Supervision. **Tobias Redlich:** Supervision, Project administration.

## Declaration of competing interest

The authors declare that they have no known competing financial interests or personal relationships that could have appeared to influence the work reported in this paper.

The author is an Editorial Board Member/Editor-in-Chief/Associate Editor/Guest Editor for *[HardwareX]* and was not involved in the editorial review or the decision to publish this article.
